# When Two Eyes Don’t Suffice—Learning Difficult Hyperfluorescence Segmentations in Retinal Fundus Autofluorescence Images via Ensemble Learning

**DOI:** 10.3390/jimaging10050116

**Published:** 2024-05-09

**Authors:** Monty Santarossa, Tebbo Tassilo Beyer, Amelie Bernadette Antonia Scharf, Ayse Tatli, Claus von der Burchard, Jakob Nazarenus, Johann Baptist Roider, Reinhard Koch

**Affiliations:** 1 Department of Computer Science, Kiel University, 24118 Kiel, Germany; tebbobeyer99@gmail.com (T.T.B.); jna@informatik.uni-kiel.de (J.N.); rk@informatik.uni-kiel.de (R.K.); 2 Department of Ophthalmology, Kiel University, 24118 Kiel, Germany; ameliebernadetteantonia.scharf@uksh.de (A.B.A.S.); ayse.tatli@uksh.de (A.T.); claus.vonderburchard@uksh.de (C.v.d.B.); johann.roider@uksh.de (J.B.R.)

**Keywords:** CSCR, central serous chorioretinopathy, fundus autofluorescence, hyperfluorescence, reduced autofluorescence, inter-observer variability, intra-observer variability, ternary, segmentation, ensemble, deep learning, U-Net, image analysis, retinal, ambiguous, annotation

## Abstract

Hyperfluorescence (HF) and reduced autofluorescence (RA) are important biomarkers in fundus autofluorescence images (FAF) for the assessment of health of the retinal pigment epithelium (RPE), an important indicator of disease progression in geographic atrophy (GA) or central serous chorioretinopathy (CSCR). Autofluorescence images have been annotated by human raters, but distinguishing biomarkers (whether signals are increased or decreased) from the normal background proves challenging, with borders being particularly open to interpretation. Consequently, significant variations emerge among different graders, and even within the same grader during repeated annotations. Tests on in-house FAF data show that even highly skilled medical experts, despite previously discussing and settling on precise annotation guidelines, reach a pair-wise agreement measured in a Dice score of no more than 63–80% for HF segmentations and only 14–52% for RA. The data further show that the agreement of our primary annotation expert with herself is a 72% Dice score for HF and 51% for RA. Given these numbers, the task of automated HF and RA segmentation cannot simply be refined to the improvement in a segmentation score. Instead, we propose the use of a segmentation ensemble. Learning from images with a single annotation, the ensemble reaches expert-like performance with an agreement of a 64–81% Dice score for HF and 21–41% for RA with all our experts. In addition, utilizing the mean predictions of the ensemble networks and their variance, we devise ternary segmentations where FAF image areas are labeled either as confident background, confident HF, or potential HF, ensuring that predictions are reliable where they are confident (97% Precision), while detecting all instances of HF (99% Recall) annotated by all experts.

## 1. Introduction

Fundus autofluorescence (FAF) imaging [1,2,3] is a fast non-invasive imaging technique that captures the natural fluorescence emitted by intrinsic fluorophores in the eye’s fundus, particularly, lipofuscin in the retinal pigment epithelium. When excited by blue light, lipofuscin releases green–yellow fluorescence, allowing for the visualization of the metabolic activity and health of the retinal cells. Areas with abnormal accumulation or loss of lipofuscin can indicate retinal pathology, making FAF a valuable tool for assessing various eye conditions. In clinical practice, fundus autofluorescence serves as a crucial diagnostic and monitoring tool for ophthalmologists. It provides insights into retinal health by highlighting changes in lipofuscin distribution, aiding in the identification and tracking of retinal diseases, such as age-related macular degeneration (AMD) and central serous chorioretinopathy (CSCR). In combination with other imaging modalities, FAF helps clinicians to establish the initial diagnosis, to assess disease progression, to plan interventions, and to evaluate treatment efficacy.

However, while the complete loss of signal in the cause of cell death leads to clearly demarked areas of no signal, distinguishing relative changes from the normal background proves challenging. While strategies like quantitative autofluorescence (qAF) [4] were developed to assess these changes more objectively, reliably annotating exact areas is difficult, with borders being particularly open to interpretation. Consequently, significant variations emerge among different graders, and even within the same grader during repeated annotations.

Especially in CSCR, areas of increased hyperfluorescence (HF) and reduced autofluorescence (RA, also hypofluorescence) are pathognomonic and can be used as a valuable diagnostic and monitoring tool. A means to reliably quantify these changes would allow for the fast and objective assessment of disease progression. Also, when combined with other imaging modalities, such as color fundus photography or optical coherence tomography, an even better understanding of a disease’s course and potentially new biomarkers seem feasible.

Artificial intelligence (AI) and convolutional neural networks (CNNs), as established in retinal image analysis [5], seem to be an obvious choice to automatically perform the segmentation of FAF images. However, they often need training data with a clearly defined ground truth by human annotators. Now, if this ground truth is subject to the above-mentioned large inter- and intragrader variability, as is often the case in medical image segmentation [6], and which was very recently shown on FAFs with inherited retinal diseases [7], this leads to the consequent dilemma that the CNN has to deal with these ambiguities.

**Figure 1:**
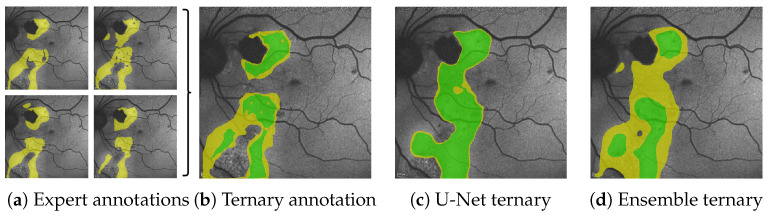
From the multiple expert’s HF annotations (yellow in (**a**)) we are able to generate segmentations for confident HF (green), where HF has been seen by all experts and segmentations for potential HF (yellow in (**b**–**d**)) where HF has been seen by some experts (see Figure 5 in Section 4.1). When comparing the expert’s ternary (**b**) to the ternary generated from a single U-Net’s prediction confidence (**c**) as well as the ternary generated from the mean prediction confidence and its variance of our proposed segmentation ensemble (**d**), we see that not only is the ensemble’s overall segmentation more accurate; the ensemble’s confident HF prediction more accurately aligns with the expert’s confident HF, whereas the single U-Net displays typical overconfidence on almost all segmented areas. Please note that the expert’s RA annotations are not shown for reasons of clarity.

In this work, we break out of this dilemma through the utilization of segmentation ensembles and bagging [8], i.e., training identical segmentation network architectures on different subsets of our training data. With this setup, even though each image in our training data features only one—potentially biased—annotation, the ensemble as a whole can accurately represent the combined and possibly diverse annotations of multiple experts. Furthermore, by leveraging the mean and variance of our ensemble prediction, we can robustly differentiate segmentations into areas of potential HF and confident HF, where a single segmentation network suffers from overconfident predictions, as shown in Figure 1.

Leakage segmentation in retinal images is an ongoing task. Some previous works have attempted automated segmentation of retinal vascular leakage in wide-field fluorescein angiography images [9,10,11]. Li et al. [12] and Dhirachaikulpanich et al. [13] use deep learning approaches to segment retinal leakages in fluorescein angiography images. However, only a few works so far have focused on the specific task of HF segmentations: Zhou et al. [14] combine diagnostic descriptions and extracted visual features through Mutual-aware Feature Fusion to improve HF segmentations in gray fundus images. Arslan et al. [15] use image preprocessing and color-maps in the HSV [16] color domain to extract HF in FAF images of patient with geographic atrophy. Their approach constitutes one of our baselines. Woof et al. [7] very recently used a single nn-U-Net [17] ensemble consisting of five U-Nets to segment, inter alia, HF, and RA in FAFs with inherited retinal diseases.

Ensemble learning has been applied to retinal image segmentation in several publications. Multiple works [18,19,20,21,22,23] have analyzed blood vessel segmentation in fundus images. Other ensemble tasks include segmentation of exudates [24,25], retinal fluids [26,27], and retinal lesions [28] in different retinal image modalities. From the above we differentiate the following two categories of ensemble learning approaches: (1) Those where identical architectures are generated differently and a final result is obtained by voting [18,21,22,24,26]; (2) Those where the elements of the ensemble fulfill separate tasks before fusing their results [22,25,27,28]. Combinations of both do, of course, exist [19,20]. Our approach falls into the first category and, as such, mimics the intuitive approach of asking for and weighing the opinions of multiple experts, reducing the likelihood of bad predictions due to an outlier prediction.

Learning segmentations from potentially ambiguous medical annotations is a known challenge. Schmarje et al. [29] use rough segmentations to identify collagen fiber orientations in SHG microscopy images by local region classification. Baumgartner et al. [30] use a hierarchical probabilistic model to derive, inter alia, lesion segmentations in thoracic CT images at different levels of resolution. Their method can be trained on single and multiple annotations. Wolleb et al. [31] utilize the stochastic sampling process of recent diffusion models to implicitly model segmentation predictions. They learn from single annotations. Rahma et al. [32] similarly use a diffusion model approach for, inter alia, lesion segmentation, but improve the segmentation diversity and accuracy by specifically learning from multiple annotations. In contrast, we use an explicit segmentation ensemble trained on single annotations. While we indeed take measures to align very fine annotation structures for segmentation consistency (see Section 2), we do not use rough labels. Available sample sizes (less than 200 training images) due to an absence of stacked scans are also lower compared to [30,31,32] whose training set sizes, e.g., for lesions range from 1k to 16k images. To the best of our knowledge, and compared to methods like [7], we are the first to explicitly approach the problem of HF and RA segmentation for ambiguous FAF annotations.

For our work, as in [33,34], we sample from the database of the University Eye Clinic of Kiel, Germany, a tertiary care center that is specialized on CSCR patients. This unique large database consists of over 300 long-term CSCR disease courses with a median follow-up of 2.5 years. Given this database and the aforementioned challenges, our contributions are as follows:

- A segmentation ensemble capable of predicting hyperfluorescence and reduced autofluorescence segmentation in FAF images on the same level as human experts. As the ensemble consists of standard U-Nets, no special architecture or training is required;
- Leveraging the inheritable variance of our proposed ensemble to divide segmentations into three classes for a ternary segmentation task: areas that are (1) confident background, (2) confident HF, or (3) potential HF, accurately reflecting the combined annotations of multiple experts despite being trained on single annotations;
- An algorithm to sample from the main ensemble a sub-ensemble of as few as five or three networks. Keeping a very similar segmentation performance to the whole ensemble, the sub-ensemble significantly reduces computational cost and time during inference;
- An additional segmentation metric $DcS_{AE}$ based on the established Dice score ( DcS ) [35] better suited to reflect the clinical reality of HF and RA segmentation, where the exact shape of a fluid prediction is less important than the fact whether HF and RA have been detected in the same area by multiple graders or not;
- Supplementary analysis of the ensemble training, from which we derive insights into optimizing the annotation approach in order to reduce costs for future endeavors in a domain, where acquiring additional training data is very costly.

## 2. Materials

To the best of our knowledge, which is supported by recent surveys [36,37,38] and research [39], there currently exists no public FAF dataset with annotated HF. Hence, like others [7,15], we can show results solely on in-house data, all of which were acquired with institutional review board approval from the University Eye Clinic of Kiel, Germany.

As in our previous works [33,34], the base of our data stems from 326 patients with CSCR, collected from 2003 to 2020. From this, we sampled patients for our retrospective study. The selected patients required a reliable diagnosis of chronically recurrent CSCR, at least three visits and a long term course of the disease of at least 2 years. Patients with an uncertain diagnosis of CSCR or an acute form of CSCR were excluded.

**Figure 3:**
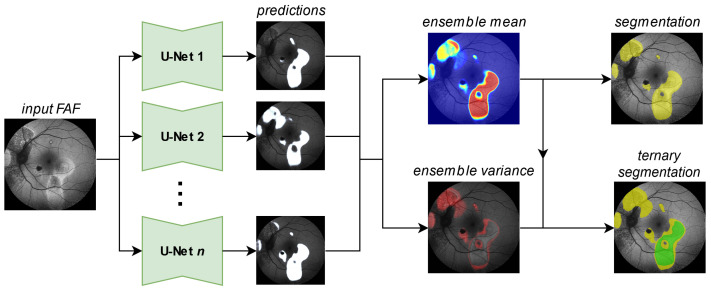
Generating a segmentation prediction (yellow HF, rest no HF) and a ternary prediction (green confident HF, yellow potential HF, rest no HF) from the proposed segmentation ensemble. Details for the generation of the ternary segmentation output are given in Figure 5.

For this work specifically, we collected patients with FAF images. FAF images are 768×768 pixels and were taken with a field of view of 30° or 55°, resulting in an average resolution of 11.3 µm/pixel or 22 µm/pixel. FAF images were acquired with the Heidelberg Retina Angiograph II (HRAII) [40]. A blue solid-state laser (wavelength 488 nm) was used to excite the fluorescein with a barrier filter at 500 nm. Image acquisition times range from 48 ms to 192 ms, the maximum Z-scan depth is 8 mm. Please note that all FAF images shown in this manuscript except for Figure 3 have a field of view of 30°.

**Figure 2:**
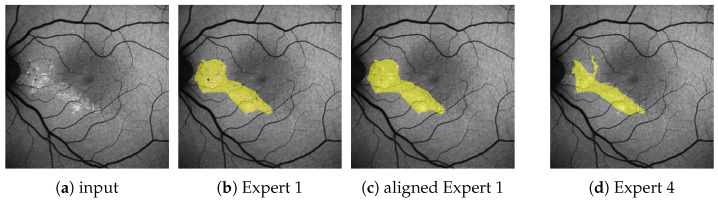
(Images are best viewed zoomed in). To align the subset of images (**a**) with fine annotations by Expert 1 (**b**) with the coarser annotations performed for the remaining images as in (**d**), we apply morphological closing ( 15×15 pixels kernel) on the segmentation masks for HF (yellow) and RF (not seen here), while ignoring granular hyper autofluorescence (violet) and granular hypo autofluorescence (blue). Should a pixel afterward belong to both the HF and the RF mask, it becomes part of the HF mask. The resulting aligned annotation is shown in (**c**).

Annotations for HF and RA are based on clinical experience and were accordingly provided by two ophthalmological experts. In order to streamline the annotation process and since initial analysis had shown considerable inter-observer variability, HF and RA were annotated coarsely, as shown Figure 2d. In total, our train split contains 180 FAF images from 100 patients. Our validation split contains 65 images of 20 patients (44 with HF, 30 with RA annotations). Our test split contains 72 images of 36 patients (60 with HF, 55 with RA annotations). Training, validation, and test data do not share any patients.

Furthermore, to analyze inter- and intra-observer variability, we utilize 9 images from 9 different patients from the validation set originally annotated by Expert 1. Additional HF and RA annotations were provided by Expert 2 and two other clinical experts, all from the same clinic, as well as Expert 1 again several months after the original annotations.

A subset of annotations by Expert 1 does not follow the aforementioned guidelines, but is significantly finer and contains the additional labels granular hyper autofluorescence and granular hypo autofluorescence. As these labels are of subordinate importance for this work, we omit them and align the finer annotations with the others by the use of morphological operations, as explained in Figure 2b,c. All results presented in this work are performed on these aligned annotations.

## 3. Methods

Table 1 provides an overview of the methods described in this section. We differentiate the classical binary segmentation task with hard labels and the ternary segmentation task (ternary for short), where areas can be marked as potential HF (or potential RA).

### 3.1. Network Ensemble

Our ensembles utilize a set of standard U-Nets (for network details see [41]) to solve the segmentation and the ternary task, as shown in Figure 3. From the Sigmoid output [42] of the individual networks predictions, we calculate the ensemble mean and variance outputs, the first of which we compare against a threshold to generate a segmentation mask. For the ternary task, we utilize both the mean and the variance output, as explained later in Figure 5. We train our segmentation ensembles separately for HF and RA with 100 networks each. Each network in our ensemble is a U-Net [43] with a ResNet34 backbone [44]. The encoder weights are pretrained on ImageNet [45].

To ensure variability between our ensemble networks, we bootstrap our training data via bagging [8] as established by previous works [18,21,24] on retinal images, i.e., by randomly sampling with replacement 152 images from our 180 training images.

We also randomly shuffle the sampled training data after each training epoch and apply random spatial augmentations (horizontal flip, shift, scale, rotation, perspective transformation) as well as pixel-level augmentations (Gaussian noise, CLAHE [46], contrast, brightness, gamma changes, sharpening, blur, motion blur) to each sample during training. No special preprocessing is applied to the input images during inference other than downscaling to 320×320 px for the U-Net. Predictions are upscaled to the original image size ( 768×768 px on our data) before all evaluations.

The segmentation networks are based on Tensorflow [47] implementations provided by [41] with augmentations from the Albumentations library [48]. Training is performed either on an NVIDIA Titan Xp GPU or an NVIDIA GTX 1070 GPU, both with an Intel Core i7-4790K CPU with 4.00 GHz. Training lasts for 200 epochs with early stopping and utilizes the binary cross-entropy loss + focal loss with the ADAM optimizer [49], a batch size of 8 and a learning rate of $1\times 10^{-4}$ .

### 3.2. Sub-Ensemble

As inference over 100 U-Nets demands potent hardware and takes a certain amount of time initializing (see Section 4.7), we propose the use of a smaller sub-ensemble to save both on memory and time. From the original ensemble of n=100 networks, we sample *m* networks for our sub-ensemble. Please note that our goal is not to accurately represent the output of the full ensemble with a smaller ensemble. Instead, it is our goal to achieve the largest diversity within our sub-ensemble to ensure variability and in effect high recall.

The process of selecting *m* elements from a set of n>m elements such that the sum of the element’s pair-wise distance is maximized is known as the MDP (maximum diversity problem) [50]. A variation of this, known as the MMDP (max–min diversity problem) [51] is concerned with maximizing the minimum pair-wise distance.

In this work, we focus on MDP. In our case, the individual networks are the elements. As distance between the elements, we define the $MSE=\frac{1}{WH}\sum_{w=1}^{W}\sum_{h=1}^{H}{\left(p_{1}\left(w,h\right)-p_{2}\left(w,h\right)\right)}^{2}$ (mean squared error) [52] between two network outputs $p_{1,}$ and $p_{2}$ over an image of width *W* and height *H*. In effect, the distance is smaller the more similar the predictions are. Specifically, we calculate for each network-pair the mean MSE over all images in the validation dataset. The result is a 100×100 distance matrix, which can be used as basis for the MDP (see Section 4.7).

For this work, we generate sub-ensembles for m∈{3,4,…,10} . To solve the MDP we use a genetic algorithm implementation in the pymoo framework [53] modified to fit our data. As baselines to the sub-ensembles achieved via MDP we consider the following configurations: (1) choosing the *m* best performing networks on the validation data. (2) Sorting by validation performance and then sampling in equal steps from that distribution (i.e., best, median and worst for m=3 ).

### 3.3. Single Networks Baselines

For comparison, we take from our proposed ensemble the single best performing U-Net (“one U-Net”), i.e., the network with the best segmentation performance on our validation dataset. Using a U-Net trained without bootstrapping was considered, but after over 10 training runs no such trained U-Net outperformed the best U-Net from the full ensemble.

Moreover, we train two additional U-Nets (“mean U-Net” and “var U-Net”), with which we can predict the ensemble mean and variance output directly (compare Figure 3) without loading any models from the actual ensemble. Both networks use a ResNet34 backbone and are trained on the mean and variance output of the full ensemble, respectively. Since this requires to regress floating point values between 0 and 1 rather than segmentation masks, we replace the previously used cross-entropy + focal loss with a mean squared error loss [52]. Training is performed for 200 epochs with the ADAM optimizer [49] and a learning rate of $1\times 10^{-4}$ . The same augmentations as for the ensemble networks are applied.

### 3.4. HyperExtract Baseline

Arslan et al. propose the HyperExtract [15] algorithm to extract and classify stages of HF regions from FAF images of patients with geographic atrophy. Taking the grayscale FAF image as input, their relevant steps for the HF segmentation task are: (1) Contrast optimization with CLAHE [46]. (2) Removal of granularity through a median filter. (3) Conversion to the JET color map [54]. (4) Cropping from 768×768 px to 700×700 px to eliminate fuzzy FAF image borders. (5) Conversion to the HSV [16] image space. (6) Extraction of HF masks through empirically determined color ranges.

In their work, Arslan et al. list color ranges for early, intermediate, and late stages of HF. As these ranges are likely dependent on the imaging device utilized to acquire the FAF data as well as the disease observed (which was geographic atrophy rather than CSCR as in this work), and since we do not differentiate between different HF stages, we conducted our own experiments to determine the optimal extraction values.

Since any color map is generated from a possible range of 256 different intensities (e.g., grayscale values, see Figure A4 in the Appendix A), we omit the color conversions (i.e., steps (3) and (5)) and instead analyze the intensity values I∈{0,1,…,255} directly. For each *I*, we calculate over our whole validation dataset how often a pixel with this value occurs after preprocessing and how often this pixel belongs to the annotated HF mask (see Figure A5). From this, we can calculate for each intensity value its probability p(I)∈[0,1] of belonging to an HF area. Via thresholding, i.e., selecting those pixels with intensity *I* such that $p\left(I\right)>=\mu_{HE}$ we can hence predict an HF segmentation.

### 3.5. Diffusion Model Baseline

Wolleb et al. [31] propose the use of a diffusion model to implicitly resemble a segmentation ensemble. Given a segmentation $x_{0}$ for an image (or stacked images) *b*, they iteratively, over *T* steps, add noise to $x_{0}$ to generate a series $x_{0}$ , $x_{1}$ , …, $x_{T}$ of successively more noisy segmentations. It is now the task of a segmentation model to predict from the concatenated inputs $x_{t}$ and *b* the less noisy segmentation $x_{t-1}$ , with t∈{1,…,T} . During training, since $x_{t-1}$ is known due to ultimately being generated from $x_{0}$ , they can calculate a loss to train the model. During inference, where $x_{0}$ is unknown, they start from random noise to iteratively predict over T steps less noisy segmentations, with the final segmentation ideally containing no noise at all. If this process is repeated for *n* different input noises, they thus generate *n* different segmentation predictions, which, however, are all informed by image(s) *b*, and hence not arbitrary.

We use the pytorch [55] implementation provided by Wolleb et al. [31] with slight modifications to fit our data. We use their original input size of 240×240 px (before cropping to 224×224 px for the network input) by downscaling our FAF images and annotations accordingly. Predicted segmentations are then upscaled during evaluation (but not during training) to fit the original image size. In their original approach, Wolleb et al. work on the BRATS2020 [56,57,58] brain MRT dataset, which for each segmentation provides four scans from different MRT series. Since we possess only single FAF images, we modified their network input to accept single layer images (i.e., *b* has shape [1, 224, 224] instead of [4, 224, 224]). We use their original T=1000 steps and learning rate of $1\times 10^{-4}$ , but have to reduce the batch size from 10 to 4 due to memory constraints. We hence trained the model longer for 100,000 instead of 60,000 iterations on an NVIDIA Titan Xp GPU. Please also note our significantly smaller training set size of 180 samples compared to the 16,298 samples used in [31]. During inference, we generate n=10 predictions.

## 4. Results

We present results regarding four different aspects of our work: (1) For the performance regarding datasets with only one available annotation, we present the segmentation and ternary scores on our validation and test data in Section 4.3 and Section 4.4, respectively. We further show pair-wise agreement among single experts and our proposed methods in Section 4.5. (2) Regarding the performance of our ternary segmentation compared to multiple accumulated expert annotations, we present results in Section 4.6. (3) For our sampling choice regarding networks for our proposed sub-ensemble, we depict the results in Section 4.7. (4) For analyzing the ensemble and network training, we show results in Sections S1 and S2 of the Supplementary.

### 4.1. Metrics

Table 2 shows the metrics use for the different tasks throughout this work, all of which will be explained in the following.

Regarding the segmentation task, an established [60] metric is the Dice score (also Sørensen–Dice coefficient, F1 score) $DcS=\frac{2|TP|}{2|TP|+|FP|+|FN|}$ [35,59], where TP , FP , and FN denote the set of true positive, false positive, and false negative segmented pixels, respectively. For ease of understanding, we keep the notation of TP , FP , and FN (i.e., by regarding the first segmentation as the prediction and the second segmentation as the ground truth).

**Figure 4:**
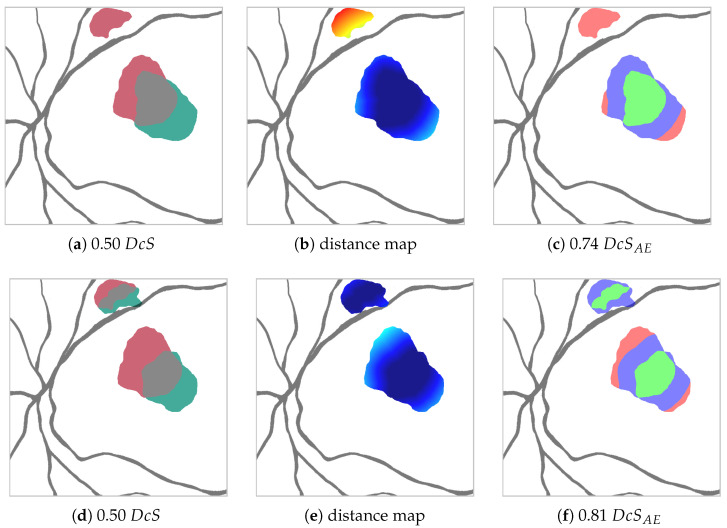
Synthetic example to show for two predictions (red and green in **a**,**d**) the difference between the Dice score DcS (**a**,**d**) and the adjusted area error Dice score $DcS_{AE}$ for $\mu_{EE}=60$ px (**c**,**f**) (TP green, EE blue, AE red) generated from the distance maps (**b**,**e**) (warmer colors indicate higher distance from agreement TP). The prediction shown in red stays the same between the top and bottom case. The prediction shown in green changes, such that on the top it only detects one red area, whereas on the bottom it detects both red areas (though the larger area with less overlap). In the context of clinical HF detection, the green prediction depicted in the bottom row is preferable to the green prediction depicted on top despite identical DcS values. The $DcS_{AE}$ metric reflects that.

While the Dice score is able to measure overall performance, it cannot necessarily depict how or why any two segmentations differ. Regarding HF and RA, we identify the following two general reasons for low segmentation scores: (1) Cases in which two segmentations agree that a biomarker is present in a certain area, but disagree over the exact outline of this area due to the absence of sharp edges; (2) Cases in which two segmentations disagree over a biomarker being present in a certain area (compare Figure 4a top and bottom).

Even though the distinction between these cases can be blurry, it is an important distinction to make. For example, the consciously perceived visual impression is generated disproportionately in the area of the fovea. Hence, retinal changes in this area are much more significant than in more peripheral locations of the retina.

This is why, given two segmentations, it is our goal to partition the set of differently labeled pixels FP∪FN into two sets EE∪AE=FT∪FN , where EEs (edge errors) is the set of pixels labeled differently due to the aforementioned case (1), and AEs (area errors) is the set of pixels labeled differently due to case (2).

In order to achieve this partition, we calculate, for each pixel in FP∪FN , its Euclidean distance to the closest pixel in TP (see Figure 4b). Should TP be empty, the distance is set to the length of the image diagonal. Pixels, whose distance is smaller or equal to the threshold $\mu_{EE}$ , are assigned to EE , the rest to AE , as shown in Figure 4c. With this, we are able to define for a given $\mu_{EE}$ an adapted Dice score $DcS_{AE}=\frac{2|TP|}{2|TP|+|AE|}$ , where, due to the aforementioned relationship EE∪AE=FP∪FN , it follows that $DcS_{AE}\geq DcS$ .

Since $\mu_{EE}$ is a sensitive parameter, we try to avoid any bias introduced by choosing a specific $\mu_{EE}$ by instead calculating a mean $DcS_{AE}$ over an equally spaced distribution of $\mu_{EE}\in \left[0,1,\ldots ,\mu_{EEmax}\right]=M_{AE}$ such that $mDcS_{AE}=\frac{1}{|M_{AE}|}\sum_{M_{AE}}DcS_{AE}$ .

**Figure 5:**
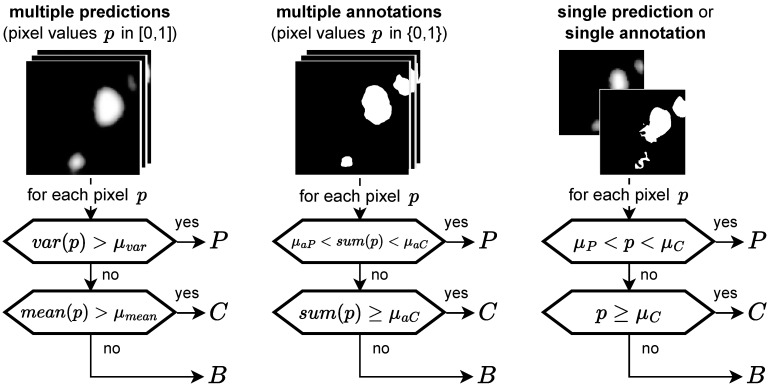
Ternary task: categorizing pixels into *P* (potential HF), *C* (confident HF), and *B* (background) depending on the input type, the general idea being that a high variance indicates *P*, whereas a low variance indicates *C* or *B*, depending on the mean. If no variance is available, two thresholds are applied to the prediction value *p*. Hence, for a single annotation no pixel can be categorized as *P*.

For the ternary task, we differentiate not only between HF and background pixels, but instead between the three categories *C* (confident HF), *P* (potential HF), and *B* (background) according to the diagram in Figure 5. Hence, when comparing two segmentations we now have, for each pixel, nine possible prediction ground truth combinations instead of the previous four (TP, FP, FN, and TN, with TN being true negative). We therefore decided to focus on the following metrics based on precision (also positive predictive value) and recall (also sensitivity) for evaluation:

(1) $$precision_{C}=\frac{|pred_{C}\cap annot_{C}|}{|pred_{C}|}$$

(2) $$recall_{CP}=\frac{|\left(pred_{C}\cup pred_{P}\right)\cap \left(annot_{C}\cup annot_{P}\right)|}{|annot_{C}\cup annot_{P}|}$$

where a high value for (1) ensures that confident predictions are definitely correct, and a high value for (2) ensures that we do not miss any HF, whether confident or potential. In addition, we utilize two more lenient but nonetheless important metrics:

(3) $$precision_{C\to CP}=\frac{|pred_{C}\cap \left(annot_{C}\cup annot_{P}\right)|}{|pred_{C}|}$$

(4) $$recall_{CP\to C}=\frac{|\left(pred_{C}\cup pred_{P}\right)\cap annot_{C}|}{|annot_{C}|}$$

where a high value for (3) ensures that confident predictions are not definitely wrong, and a high value for (4) ensures that we do not miss a confident annotation.

Though it does not happen in our case, it must be noted that the metrics presented above can be bypassed by predicting *every* pixel as *P*. The result would be undefined for (1) and (3), and a perfect score of 1one for (2) and (4). We hence also observe

(5) $$precision_{CP}=\frac{|\left(pred_{C}\cup pred_{P}\right)\cap \left(annot_{C}\cup annot_{P}\right)|}{|pred_{C}\cup pred_{P}|}$$

from which, together with Equation (2), we calculate a Dice score as a sanity check:

(6) $$DcS_{CP}=\frac{2\cdot precision_{CP}\cdot recall_{CP}}{precision_{CP}+recall_{CP}}$$

In the case where we apply the ternary task to images with just one expert annotation, we note that (1) = (3) and (2) = (4) since $|annot_{P}|=0$ according to Figure 5.

Please also note that for the ternary task, we do not calculate EEs or AEs. The reason for this is the fact that we did not find it to provide much further insight compared to $mDcS_{AE}$ already given for the segmentation task, which we report on each dataset.

### 4.2. Parameter Selection

For evaluation, we chose the following values for our parameters. For the $mDcS_{AE}$ metric, $\mu_{EEmax}=100$ px was set as that high a value made it sure that all instances would be covered where differences in predictions could reasonably be interpreted as an EE. Measurements of EEs among experts, as later depicted in Figure 8 in Section 4.5, support these observations.

**Figure 6:**
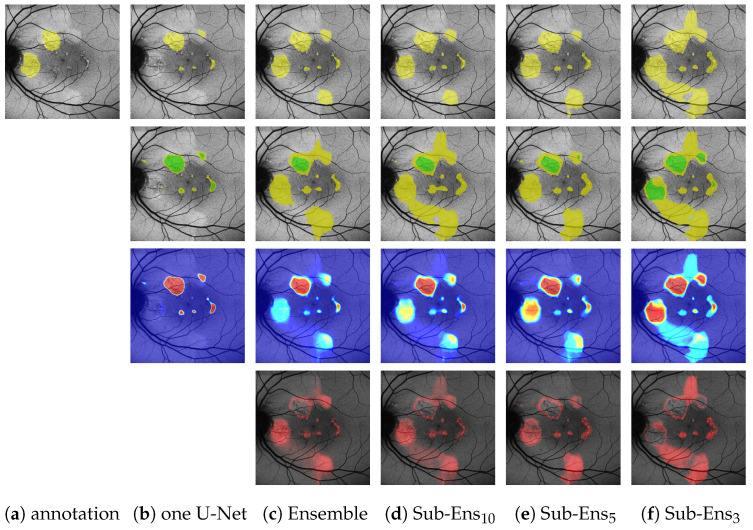
(Images are best viewed zoomed in). HF segmentation predictions (top row), ternary predictions (second row), prediction means (third row), and variances (bottom row) for an image in the validation dataset. For the ternary task, yellow indicates *P* (potential predictions) and green *C* (confident predictions). Sub-Ens*_m_* depicts the prediction of a sub-ensemble with *m* networks.

Regarding the ternary task, for annotations we set $\mu_{aC}=5$ in order to classify an area as *C* only if all five experts segmented it. $\mu_{aP}=2$ was set, as review with the graders revealed that occasionally single graders mislabeled areas, e.g., of background fluorescence as HF. Hence, an area is classified as *P* if not all, but at least two experts segmented it. For our ensemble’s predictions, we set $\mu_{mean}=0.2$ following the logic that, similar to the experts, more than 1/5th of our ensemble needed to confidently detect it. $\mu_{var}$ was set empirically to 0.1 . For single predictions or annotations, we set $\mu_{C}=0.9$ and $\mu_{P}=0.1$ . From confidence maps as, e.g., depicted in the second-lowest row in Figure 6b, we inferred that these parameters are not very sensitive, as predictions of a single network tend to be very close to either 0 or 1 with small edges between these two extremes. For HyperExtract, we chose $\mu_{HE}=0.07$ as this is the optimal value for the validation set (see Figure A6).

### 4.3. Segmentation and Ternary Performance—Validation Set

Table 3 and Table 4 depict segmentation and ternary scores for HF and RA on the validation dataset, respectively.

Comparing a single U-Net to the proposed ensemble, we see that for the classical segmentation task we marginally lose performance (from 0.668 to 0.677 DcS on HF|from 0.497 to 0.501 on RA). For the $mDcS_{AE}$ score, where the edge error is heavily suppressed, we see a very comparable margin for HF (from 0.825 to 0.814) and even a significant improvement for RA (from 0.677 to 622). We can hence infer that this difference in performance is not mainly caused by EEs (i.e., unsharp edges), but indeed due to new areas being segmented. Figure 6b,c (top row) shows an example of this.

However, when looking at the ternary task performance, we see that, in regard to $precision_{C}$ and $recall_{CP\to C}$ , our proposed ensemble drastically outperforms the single U-Net. Especially the improvement for $recall_{CP\to C}$ (from 0.872 to 0.804 on HF|from 0.754 to 0.512 on RA) has to be noted, indicating that the ensemble is detecting far more of the annotated HF (at the cost of some potential overpredictions, as depicted by 0.035 lower $DcS_{CP}$ ). This again is shown by Figure 6b,c (second row), where the ensemble, in contrast to the single U-Net, is able to detect the leftmost HF area to the immediate right of the optic disc. Furthermore, the depicted images also reflect the differences in $precision_{C}$ : The ensemble is only confident in one part of the two segments annotated by the expert, whereas the single U-Net is also highly confident in image areas that have not been annotated by the expert.

Looking at the sub-ensembles, we note that for the segmentation task and $precision_{C}$ they perform slightly worse than the full ensemble, scores getting closer the more networks we include. However, regarding $recall_{CP\to C}$ , the sub-ensembles especially for RA perform even better than the whole ensemble. We also want to highlight the fact that even the minimal sub-ensemble with three networks significantly outperforms the single U-Net for $recall_{CP\to C}$ (from 0.868 to 0.804 on HF|from 0.662 to 0.512 RA).

Regarding the mean U-Net, we see competitive results on HF, but not on RA. The combination of a mean and a variance network drastically decreases performance on both HF and RA. H-Extract is not competitive and tends to generate scattershot segmentations despite optimized extraction values, as visible in Figure A7. Analyzing the relationship between HF and pixel intensities, as performed in Figure A5, reveals that on our FAF data, pixel intensity is not a good indicator for HF except for very high intensities, which, however, only appear very rarely. The diffusion model, despite some good segmentations as seen in the bottom row of Figure A8 does, in other cases, not predict anything (top row of the same figure). It is also susceptible to small changes, as seemingly similar images from the same eye lead to very different predictions, as shown in the middle row of Figure A8.

### 4.4. Segmentation and Ternary Performance—Test Set

Table 5 and Table 6 depict the segmentation and ternary scores on the test set for HF and RA, respectively. Notably, the results for all methods are lower than on the validation set. This is true even for the diffusion model, which was not optimized for the validation set, indicating that the test set is more dissimilar to the training set than the validation set. Since the possible optimal score for HyperExtract, as shown in Figure A6, is also significantly lower on the test than the validation, the indication might be that the test set is overall more diverse and hence more challenging to segment.

Still, the general observations from the validation dataset hold true. We want to point out the fact that the ensemble significantly outperforms the single U-Net on all metrics (except $DcS_{CP}$ on RA, where it is 0.002 worse).

Regarding the sub-ensembles, we observe Sub-Ens_10_ to perform even better than the ensemble on $recall_{CP\to C}$ (from 0.631 to 0.606 on HF|from 0.403 to 0.631). The mean U-Net, similar to the results on the validation dataset, performs comparatively well on HF, but not on RA data. The combination mean + variance U-Net again fails to improve the results on both pathologies. The H-Extract results are not competitive, despite the chosen $\mu_{HE}=0.07$ being close to the optimum for the test dataset (see Figure A6). Neither is the diffusion model, the results being lowest among all methods.

**Figure 7:**
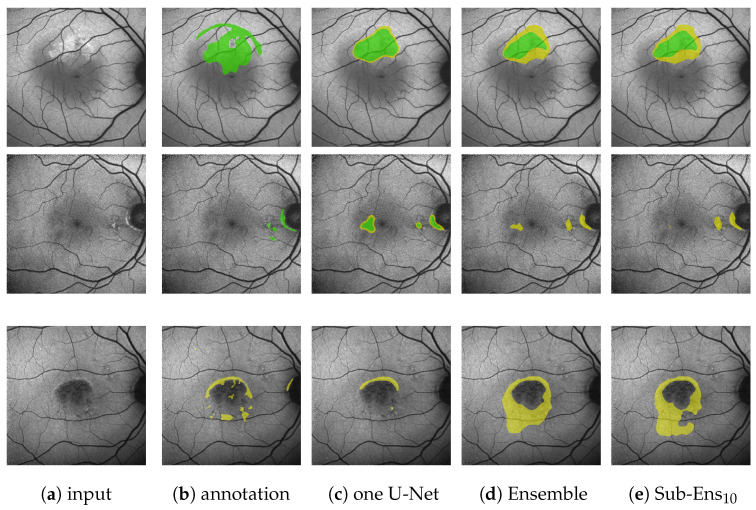
(Images are best viewed zoomed in). HF prediction results on the test set for our proposed ensembles (**d**,**e**) and a single segmentation U-Net (**c**). Images (**a**) where chosen such that the results for Sub-Ens_10_ (0.67 $recall_{CP\to C}$ top, 0.59 $recall_{CP\to C}$ middle, 0.66 $mDcS_{AE}$ bottom) are close to the mean results (0.63 $recall_{CP\to C}$ , 0.63 $mDcS_{AE}$ ) shown in Table 5. Please note that the upper two rows show results and annotations (**b**) for the ternary task, while the bottom row shows results and annotations (**b**) for the segmentation task.

**Figure A1:**
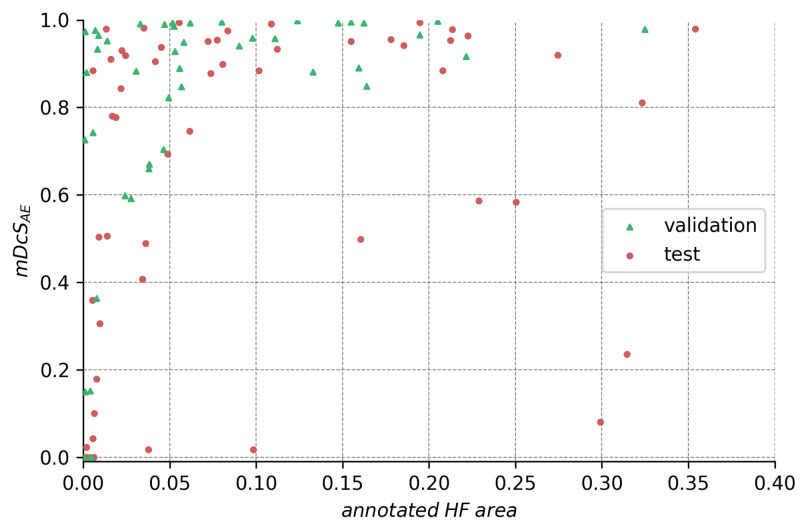
HF segmentation performance in relation to the size of annotated HF for the ensemble.

**Figure A2:**
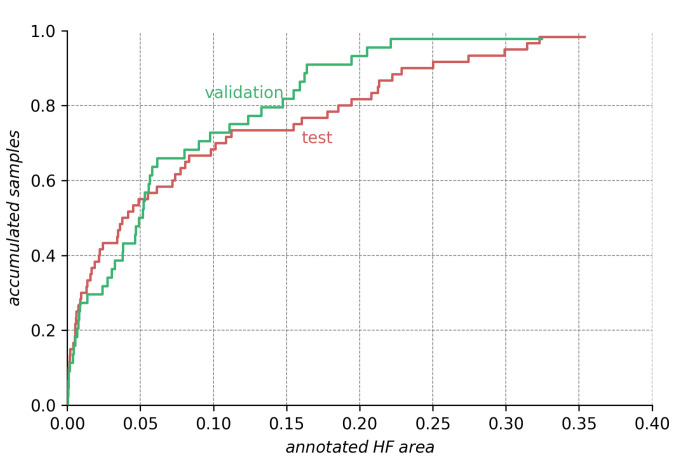
Comparison of HF annotation sizes for the test and validation dataset. The height of the curve for any given annotation size on the abscissa indicates the ratio of *smaller* annotations in the dataset. We see that the test dataset contains more extreme (very small and very large) HF annotations than the validation dataset.

**Figure A3:**
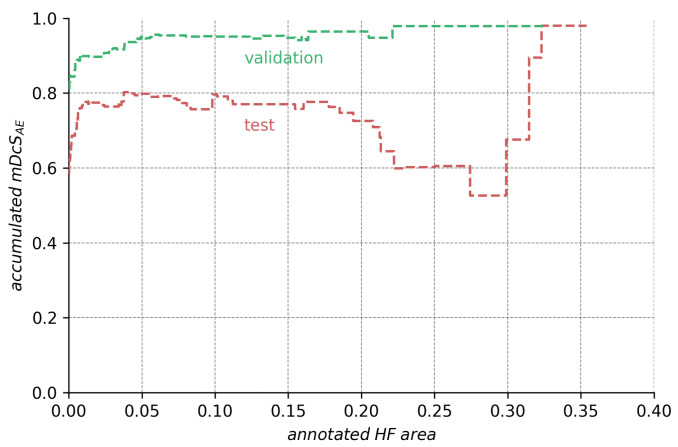
The ensemble’s segmentation score in regard to the annotation size. The height of the curve for any point on the abscissa indicates the $mDcS_{AE}$ score the ensemble would obtain if we only considered the images with larger HF annotations. The data shows that lower test scores are caused mainly by bad segmentations for a few images with very large HF area (compare Figure A1). The data also show that segmentation scores are generally high but suffer from the set of images with very small annotations.

Analyzing the ensemble’s expected performance on the test dataset, we see from Figure 7 that the average results for the ensembles are still acceptable. To investigate the factors contributing to the disparity between the test and validation performance, we analyzed those differences in regard to HF annotation size in Figure A1, Figure A2, Figure A3 in the Appendix A. From the data depicted, we see that the decreased performance stems (1) from the test set’s higher ratio of samples with very small HF annotations (<5% image size), but also (2) from bad performance on a few images with large annotations (>20% image size).

### 4.5. Segmentation Performance—Agreement among Experts and Ensembles

Table 7 and Table 8 show the agreement among all five available expert annotations for nine FAF images as segmentation scores. Looking at DcS alone, we see that the experts only have an agreement of from 0.63 to 0.80 (mean 0.69) for HF and from 0.14 to 0.52 (mean 0.36) for RA.

However, significantly higher $mDcS_{AE}$ scores (mean 0.82 for HF|0.48 for RA) indicate that the comparatively low DcS scores are, in many cases, caused by edge errors and not—which would be clinically relevant—by area errors, i.e., differently seen locations of HF and RA.

**Figure 8:**
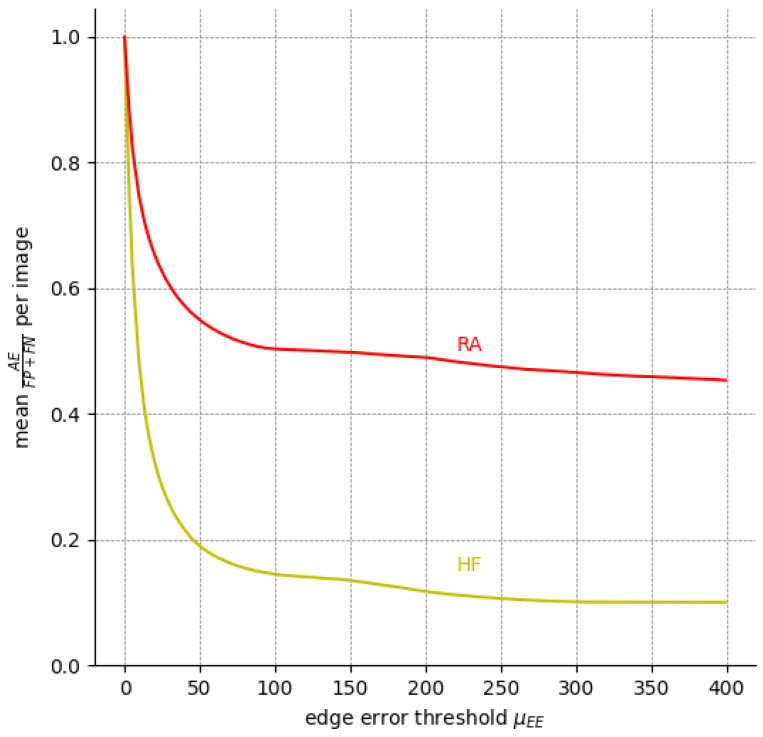
The ratio of AE compared to FP+FN (i.e., all pixels labeled differently) among the five available expert annotations over nine FAF images for different $\mu_{EE}$ . The ratio of EE is the inverse of the curve depicted here since EE+AE=FP+FN .

We can infer the same from Figure 8, which depicts the percentage of EEs and AEs among all expert annotation pairs for possible values of the edge error thresholds $\mu_{EE}$ . Evidently, EEs outweigh AEs for a threshold as low as $\mu_{EE}=10$ px for HF. For $\mu_{EE}=50$ px, AEs already make up less than 20% of HF the errors, whereas RA is a more difficult label.

**Figure 9:**
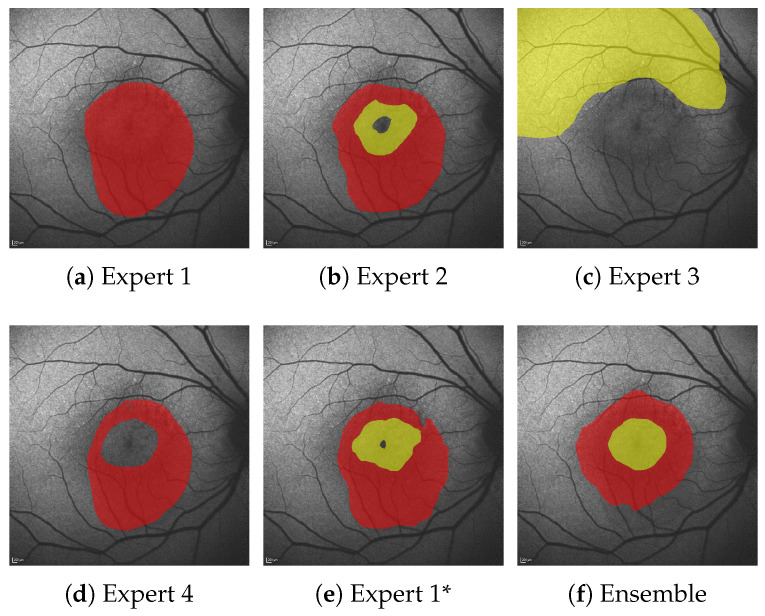
Comparison of experts annotations (**a**–**e**) and ensemble predictions (**f**) for HF (yellow) and RA (red). Expert 1* denotes segmentations of Expert 1 several months after the original annotations.

Still, at least 8% of HF errors are due to cases where no pair-wise overlap between the expert annotations exists, and are hence an AE by default. Figure 9 shows one such case where significant AEs are present, even among expert annotations.

Regarding the performance of the AI-driven methods, we note that for HF, all of them reach scores comparable to the experts ( $mDcS_{AE}$ 0.87 for ensemble|0.86 for Sub-Ens_10_|0.86 for mean U-Net|0.84 for single U-Net). However, on RA, the ensembles perform noticeably better than the single U-Net ( $mDcS_{AE}$ 0.47 for ensemble|0.47 for Sub-Ens_10_|0.45 for mean U-net|0.38 for single U-Net), reflecting the previous results on the validation and test data. The mean U-Net, on the other hand, performs surprisingly well on RA, given the previous results.

### 4.6. Ternary Performance—Comparison against Accumulated Expert Annotations

Table 9 and Table 10 illustrate the ternary performance on the ground truth from multiple experts for HF and RA, respectively. We see that the ensemble and sub-ensembles perform better than a single U-Net on all of the four main metrics.

**Figure 10:**
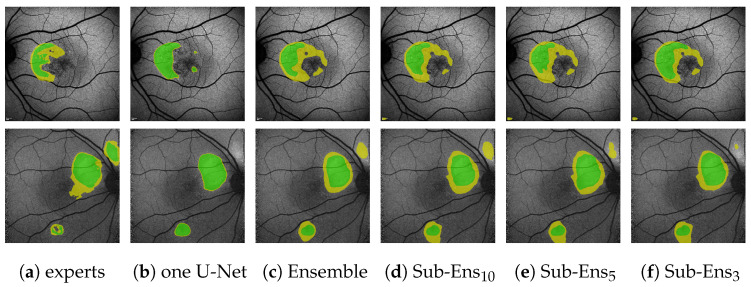
(images are best viewed zoomed in). HF ternary performance for different ensembles (**c**–**f**) and the single U-Net baseline (**b**) on two images. “experts” (**a**) denotes the ternary ground truth created from all 5 available expert annotations.

Of notable significance are the ensemble’s improvements over a single U-Net for $recall_{CP}$ (0.88 compared to 0.79) and $recall_{CP\to C}$ (0.99 compared to 0.96). Especially the latter score is important, as it shows that on the given data we detect *every* instance of HF annotated by all experts, whereas a single U-Net does not. An example of this can be seen in the bottom row of Figure 10, where the single U-Net, despite a very confident prediction, misses a significant HF area above the optic disk, which was indeed annotated by all experts. The ensemble, while not confident, does detect that very same area.

Though we are careful not to draw too decisive conclusions from only nine images, we do point attention to the fact that the ensemble’s $recall_{CP}$ score of 0.88 is very comparable to aforementioned results on the validation dataset (0.87 as reported in Table 3; note that on the validation set $recall_{CP}$ = $recall_{CP\to C}$ ).

Regarding the sub-ensembles, we infer from Table 9 that especially the sub-ensembles with 10 and 5 networks are very close in performance to the full ensemble. This is supported by the visual examples in Figure 10. Here, e.g., in the top row, the ensemble and all sub-ensembles robustly, and similarly to some experts, segment HF above and below the fovea (as well as almost encircling it to the right, contrary to the expert annotations), while the single U-Net, albeit confident, only predicts a large area of HF to the left of the fovea.

From this example, as well as from the $DcS_{CP}$ scores in Table 9, we see that the improved ternary scores for the sub-ensembles again come at the cost of slight overpredictions.

Regarding the RA predictions given in Table 10, we note that the precision scores have to be interpreted very carefully due to little to no area being actually predicted as *C*. This explains missing and seemingly perfect precision scores. The $recall_{CP}$ scores, however, do show that the proposed ensemble and sub-ensemble do detect potential RA with significantly more reliability than the single U-net approaches (from 0.57 to 0.68 for ensembles vs. a maximum of 0.31 for the single U-Nets). Notably, Sub-Ens_10_ again detects more considerably more RA (0.68 to 0.59 $recall_{CP}$ ) at the cost of some overpredictions (0.45 $DcS_{CP}$ vs 0.55 $DcS_{CP}$ ).

### 4.7. Sub-Ensemble Comparison

**Figure 11:**
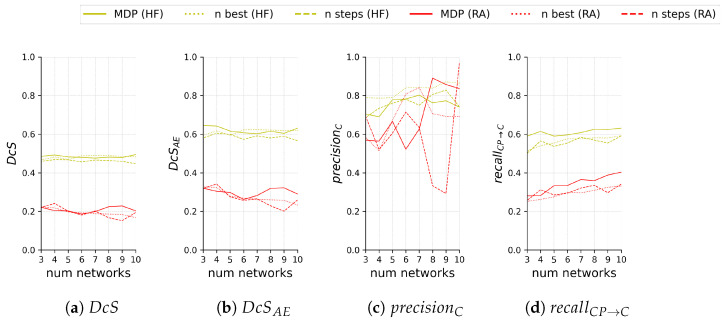
Segmentation scores (**a**,**b**) and ternary scores (**c**,**d**) on the test set of 60 images with HF (yellow) and 55 images with RA (red) for the proposed sub-ensemble sampled by solving the MDP and the sub-ensemble baselines sampled either by selecting the *n* best performing networks (*n* best) or sampling in equal steps (*n* steps) based on the segmentation performance (i.e., worst, median, and best for n=3 ). Scores are shown in relation to the number *n* of networks in the sub-ensemble.

Figure 11 compares the test segmentation and ternary scores for the different sub-ensemble sampling approaches both on HF and RA depending on the number of models included in the sub-ensemble.

We see that our proposed method performs very comparably for DcS and $mDcS_{AE}$ on both HF and RA, despite our sampling not being optimized for segmentation scores. Regarding $recall_{CP\to C}$ , our proposed sub-ensemble performs significantly better on both modalities for all numbers of models except for four models on RA, where it is second best.

A direct comparison for $precision_{C}$ is difficult if the area of *C* is not taken into account. This is especially notable for results on RA, where for seven or more models all sub-ensembles see very drastic changes, both positive and negative. For HF, sampling the best *n* models for the sub-ensemble performs consistently best.

Table 11 compares the inference times for our proposed ensemble and sub-ensembles. An initial cost in time is required to load the models and for predicting the first image. Afterwards, due to caching and optimizations by Tensorflow, the inference times are significantly reduced. Notably, sequential inference over the whole ensemble takes less than 4 s per image. Not explicitly included in this are the time it takes to load a single image, push it to the GPU, and receive the ensemble’s prediction, since all these operations are parallelized during batch prediction. Hence, the inference time for a predicting single image as it might occur in clinical practice could be slightly higher.

Also given are the inference times for a single forward pass of the diffusion model (i.e., the time needed to generate for one FAF image a number of segmentations equal to the batch size). We report no separate time for the first image, since the diffusion model itself is applied T=1000 times per single HF prediction due to the iterative denoising approach. We note that our full ensemble is faster by a factor of over 16 compared to the diffusion model. Should GPU limitations only allow for a batch size smaller than the desired number of segmentations per FAF image, this factor multiplies by $\left\lceil \frac{\mathrm{num}\;\mathrm{segmentations}}{\mathrm{batch}\;\mathrm{size}}\right\rceil$ .

Comparing memory requirements the same way as runtimes is difficult due to dynamic memory allocation and serialization, but we note that while both the NVIDIA Titan Xp GPU and the NVIDIA GTX 1070 GPU could run all sub-ensembles proposed in this work, only the Titan Xp GPU could run the whole ensemble in parallel.

## 5. Discussion

### 5.1. Inter-Grader Agreement

From our analysis, we see that HF and particularly RA are difficult labels to segment precisely. Our mean results of 0.69 DcS agreement on HF and 0.36 DcS on RA are partly in accordance to the numbers recently reported by [7], who on FAFs with inherited retinal diseases had an inter-grader agreement of 0.72 DcS for HF and 0.75 DcS for RA.

Their higher RA agreement can be explained by their definition of RA having to be at least 90–100% as dark as the optic disc [7]. If we compare this to our nine images with multiple annotations, we see that if we calculate the relative darkness as $\frac{1-\mathrm{mean}\;\mathrm{intensity}\;\mathrm{RA}}{1-\mathrm{mean}\;\mathrm{intensity}\;\mathrm{optic}\;\mathrm{disc}}$ our expert’s annotated RA being only 71–85% as dark as the optic disc.

### 5.2. Area Error Dice Score

The introduction of $mDcs_{AE}$ was very helpful in distinguishing disagreements over the exact shape of HF from the much more important case where two graders disagree over the presence of HF in certain image areas. While for the most part in our evaluation, due to similar architectures close DcS values do indicate close $mDcS_{AE}$ values, there are cases where this is not the case, e.g., Sub-Ens_3_ vs. Sub-Ens_5_ in Table 5 or most notable in Figure S2 in the Supplementary for *max* sampling.

We also think that presenting $mDcS_{AE}$ over $DcS_{AE}$ for some specific threshold avoids a selection bias. This comes at the cost of making the metric less intuitive to visualize, i.e., it is not as clear as with DcS or intersection over union (also Jaccard index) [61] what a specific value of $mDcs_{AE}$ looks like.

It should also be noted that, in its current state, $mDcs_{AE}$ could be expressed as linearly weighing each FP and FN pixel based on its distance to the nearest TP pixel (or, more precisely, the minimum of its distance and the threshold $\mu_{EEmax}$ ). In the future, other weighing functions might be found even more useful.

### 5.3. Ternary Task

Our approach of utilizing a ternary segmentation system in contrast to classical binary segmentation has shown to be useful on the available data. Providing an additional label of “potential segmentation” allows us to handle the difficult task of HF and RA segmentation by keeping a high precision for confident predictions (more than 97% of confident predictions seen by at least two experts) and a very high recall (99% of HF annotated by all experts found).

A current limitation in our analysis is the fact that we do possess only very few images with multiple annotations, somewhat limiting our evaluation on the benefits of the ternary system. Future work on larger FAF datasets with multiple annotation is desired.

### 5.4. Ensemble Segmentation

We could show the benefit of using an ensemble over a single network repeatedly on all datasets, especially in regard to the ternary task on annotations from multiple experts. This is all the more notable as our training data stems from annotation data of just one expert. Future work might analyze how comparatively low agreement of the expert with themselves (0.85 $mDcS_{AE}$ , mean 0.82 $mDcS_{AE}$ for all experts among each other) might have helped in avoiding overfitting and creating diverse ensemble networks.

Our sampling approach for the sub-ensembles aiming at maximum diversity is well suited, as all sub-ensembles perform consistently well on all datasets. With as little as five networks (1/20th) we are within 1% of the full ensemble’s performance on three of the four main ternary metrics (and within 3% for $precision_{C}$ ). For 10 networks, we achieve equal or even higher recall values than the full ensemble on all datasets, indicating that this particular sub-ensemble entails a good set of varied models, which, in the full ensemble, are partly suppressed by the majority of more similar networks. Considering these achievements, we think the occasional and slight overprediction (0.05 and 0.06 lower $DcS_{CP}$ for HF) to be very acceptable. Additional evaluation, detailed in Section S3 of the Supplementary, shows that our segmentation U-Nets are generally robust against image noise.

We see a limitation in the fact that we currently cannot systematically set up diverse networks before or during training (compare Section S1 in the Supplementary). While bootstrapping is evidently capable of creating sufficiently diverse networks from annotations of just one expert to avoid overfitting and overconfident predictions, we currently have little control over the process of how to generate this diversity. The similarity or dissimilarity of the selected training data or patients between networks gives little indication for the similarity of the output predictions. This necessitates the time and hardware resources for the training of many networks in the hope that we can select a suitable subset afterward. Future work hence needs to look at the influence of the other random parameters (random initialization, augmentation, data shuffling, GPU parallelization) in order to allow the selected training of diverse networks.

Future work will also analyze on how well our ensembles generalize to new data, e.g., data of other centers or from other FAF imaging devices. While we do not apply any data-specific preprocessing, but do use strong augmentations during training and hence would expect some robustness to new data, we do note that further evaluation is required.

In this regard, we would also like to point out that our proposed sub-ensemble sampling-approach based on diversity does not require ground truth. This opens up the possibility of selecting from a set of models trained on one dataset a subset with maximum diversity on another dataset without the need for further annotation.

### 5.5. Baselines

**Figure A4:**
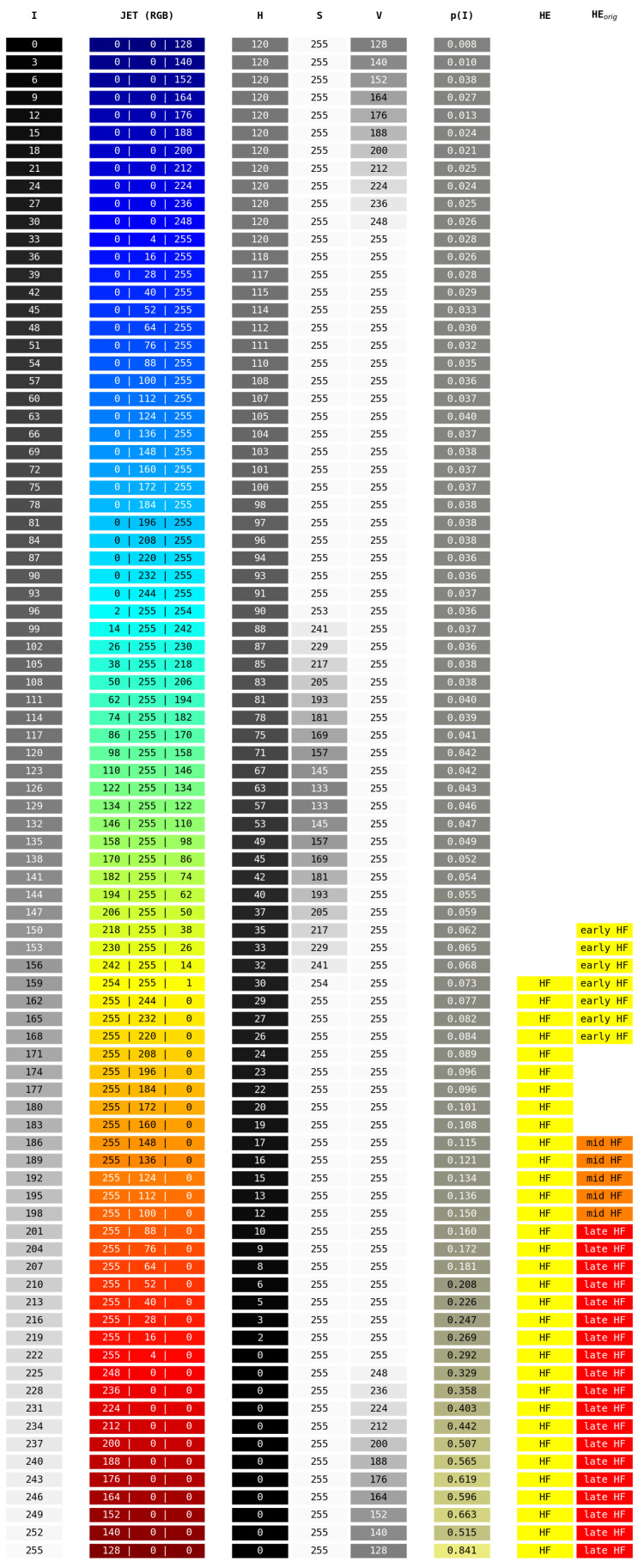
The HyperExtract [15] color conversion table. Given are the image’s grayscale intensity values I after preprocessing (with step size 3 for readability), the resulting JET color map RGB values and resulting HSV values. HSV values are given in the ranges of the OpenCV library [66], as [15] explicitly mention the usage of the OpenCV *inRange()* function. p(I) denotes the probability of a pixel with intensity *I* after preprocessing to be HF in our validation dataset. The column *HE* denotes pixels that will be classified as HF by our adapted HyperExtract method. The column *${\mathrm{HE}}_{orig}$* denotes pixels that will be classified as different stages of HF by the original extraction ranges given in [15].

**Figure A5:**
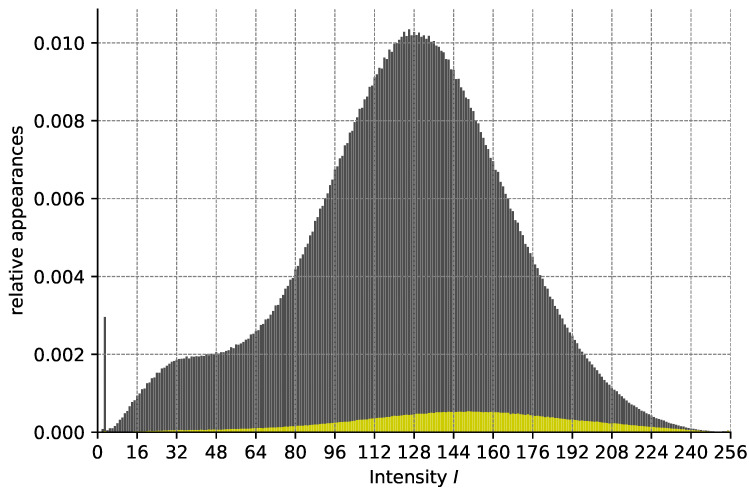
Analysis of our validation dataset in regard to the relationship of pixel intensity *I* after preprocessing and the probability of belonging to an HF area. The height of the gray bars (from 0) depicts the relative number of appearances of pixels with a given intensity I. The height of the yellow bars depicts the relative number of pixels with this intensity belonging to HF. Pixels with intensity 0 are omitted for better readability.

**Figure A6:**
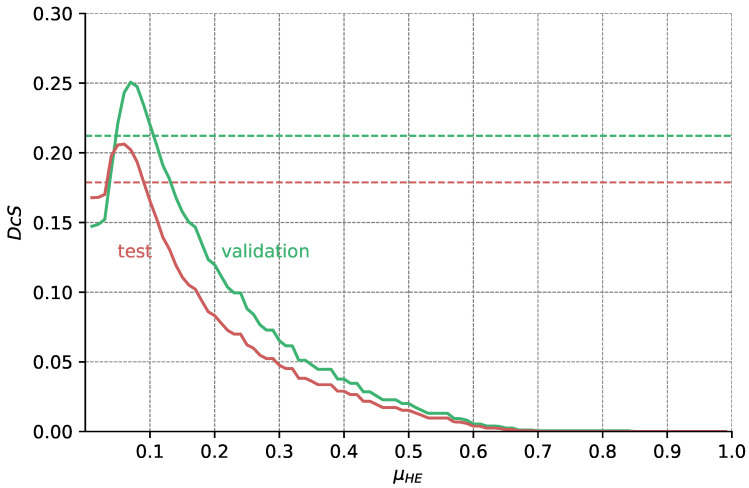
Validation and test Dice scores for the HyperExtract baseline over different thresholds $\mu_{HE}$ . From the data shown in Figure A5, we calculate for each pixel intensity *I* its probability p(I) of belonging to HF (as depicted in Figure A4). Only pixels with $p\left(I\right)>=\mu_{HE}$ are segmented as HF. Optimal results for the validation and test dataset are achieved with $\mu_{HE}=0.07$ and $\mu_{HE}=0.06$ , respectively. The dashed horizontal lines show the results achieved with the original extraction ranges given in [15].

**Figure A7:**
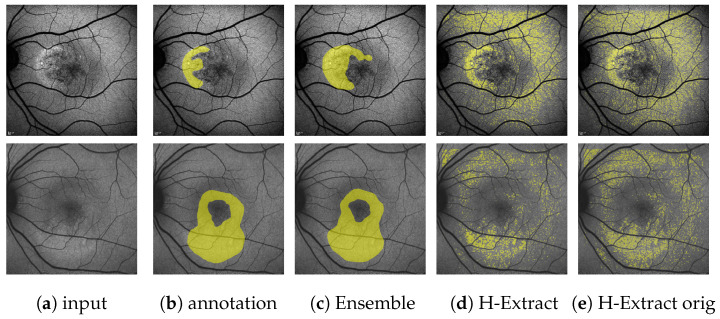
(Images are best viewed zoomed in). HF segmentations on the single-annotation validation set for HyperExtract [15] both for our adapted method (**d**) and the method with the original extraction ranges (**e**). Predictions of our full ensemble (**c**) and the expert annotations (**b**) are given for comparison. The top row depicts the same image, as shown on the top in Figure 10, the bottom row shows an image, where the H-Extract achieves a DcS=0.255 very close to its mean DcS on the validation set (=0.251, see Table 3).

**Figure A8:**
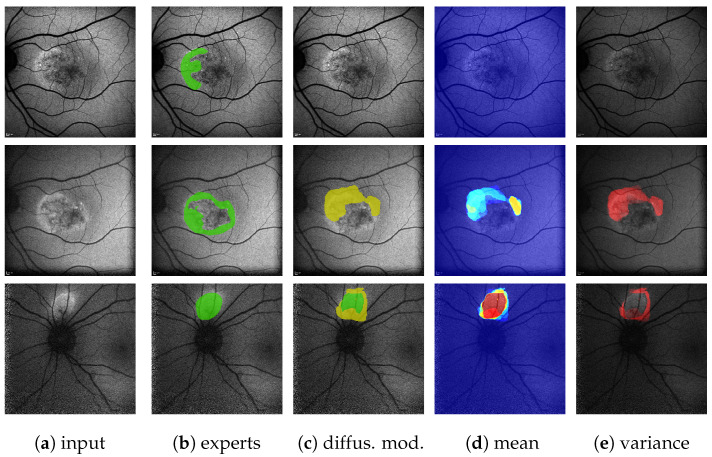
(Images are best viewed zoomed in). HF ternary predictions on the single-annotation validation set for the diffusion model baseline [31] (**c**) with 10 predictions per image plus mean (**d**) and variance (**e**) outputs. The top row shows the same image as on the top of Figure 10 and Figure A7. Here, the diffusion models in 10 predictions dos not predict anything. The middle row shows the same eye as the top but at a later date. Here, the diffusion model reaches $recall_{C\to CP}=0.466$ close to its mean on the validation set (=0.456, see Table 3). The bottom row shows a case where the diffusion model reaches a very good score of $recall_{C\to CP}=0.952$ .

Regarding HyperExtract [15], we showed that the color conversion proposed in the original paper brings no benefit over working directly on the grayscale values (Figure A4). Still, even with our optimization approach i.r.t. to the probability of a pixel intensity indicating HF, the prediction performance is not competitive. We hence conclude that on our data, HF segmentation based solely on pixel intensity does not yield sufficient results.

Regarding the diffusion model by [31], we note that even with our very limited training data compared to the original work, we occasionally generate very good results, indicating that with sufficient amounts of data, these more expressive models might be able to generate good HF segmentations. Still, while having to train only a single network for multiple predictions is beneficial, we notice drawbacks both in the fact that we have little to no control over the diversity of the output due to feeding random noise into the algorithm and in that the denoising process takes considerably longer, which inferences our proposed segmentation ensembles.

### 5.6. Runtimes

The runtimes of our proposed models (less than 1/3 of a second for all sub-ensembles) are very suitable for a clinical setting. It is notable that even the whole ensemble with 100 models is able to predict an image in roughly 3 s. We believe this to be acceptable for clinical application, where a typical use case would encase loading the model once, and then providing predictions as a microservice, hence rendering the drawback of a long initial loading time of up to 6 min almost irrelevant. Optimizations and parallelization could further reduce this latency, though we consider these out of scope for this work.

We also note that we are significantly faster than the diffusion model baseline by a factor of at least 16 for the full ensemble and by a factor of at least 181 for the sub-ensemble with 10 models.

### 5.7. Annotation Selection

While the results of our current analysis (shown in Section S2 of the Supplementary) are interesting in the sense that a number of averaged size annotations seem to yield better results than an equal number of mixed or large annotations, we do note that further evaluation is necessary to derive general annotation guidelines from this. One aspect that should be analyzed in future work are the properties of the validation and test set, though we see, e.g., from Figure A2, our current validation set does contain a mix of small, medium and large samples.

Other factors besides annotated area were considered as well (e.g., number of individual patients vs. data from multiple appointments of the same patient), but due to limitations in our current set of annotated data, they could not be sufficiently analyzed.

Now that we possess an initial dataset capable of training a segmentation ensemble, the integration of active learning aspects as in [62] to specifically target annotation ambiguity could prove to be useful.

## 6. Conclusions

Our proposed segmentation ensemble is able to robustly predict difficult areas of hyperfluorescence (HF) in fundus autofluorescence images. On data annotated by multiple experts, we achieve the same agreements with the experts as the experts among themselves both for HF and reduced autofluorescence. Furthermore, on data annotated by a single expert, our proposed ensembles outperform single networks as well as contemporary approaches for HF segmentation based on pixel intensity.

Our proposed method of sampling models for sub-ensembles based on dissimilar predictions is able to keep high recall values for HF and RA prediction, occasionally even improving over the whole ensemble, while significantly reducing the required time and memory cost for predictions.

## Figures and Tables

**Table 1:** List of proposed methods (†) and baselines as well as their targeted applications. Checkmarks in brackets denote methods that have been applied to the ternary task for completeness, but, due to the lack of available variance data, need a different approach to differentiate confident from potential predictions (compare Section 4.1). * are only applied to HF and not to RA.

Method	Detailed in	Segmentation Task	Ternary Task
Ensemble (†)	Section 3.1	✓	✓
Sub-Ensemble (†)	Section 3.2	✓	✓
one U-Net	Section 3.3	✓	(✓)
mean U-Net	Section 3.3	✓	(✓)
mean + var U-Net	Section 3.3	-	✓
HyperExtract [15] *	Section 3.4	✓	(✓)
Diffusion Model [31] *	Section 3.5	✓	✓

**Table 2:** Overview over metrics used in this work depending on the task and the number of available experts annotations.

Task	Available Annotations	Used Metrics
segmentation	one	DcS (Dice score) [35,59], $mDcS_{AE}$
ternary	one	$precision_{C}$ , $recall_{CP\to C}$ , $DcS_{CP}$
ternary	multiple	$precision_{C}$ , $recall_{CP\to C}$ , $precision_{C\to CP}$ , $recall_{CP}$ , $DcS_{CP}$

**Table 3:** Segmentation and ternary scores for HF on the single-annotation validation set of 44 FAF images. Best scores are drawn bold and underlined, second-best scores are drawn bold, ″ indicates scores are the same as for mean U-Net.

	Segmentation Task		Ternary Task		
	DcS	${\mathrm{mDcS}}_{\mathrm{AE}}$	${\mathrm{precision}}_{C}$	${\mathrm{recall}}_{\mathrm{CP}\to C}$	${\mathrm{DcS}}_{\mathrm{CP}}$
**Ensemble**	**0.668**	**0.814**	**0.889**	**0.872**	**0.642**
**Sub-Ens_10_**	0.663	0.806	0.848	**0.881**	0.600
**Sub-Ens_5_**	0.638	0.787	0.802	0.863	0.603
**Sub-Ens_3_**	0.628	0.763	0.786	0.868	0.621
**mean U-Net**	0.640	0.779	**0.951**	0.830	0.635
**mean + var U-Net**	″	″	0.777	0.748	0.609
**one U-Net**	**0.677**	**0.825**	0.780	0.804	**0.668**
**H-Extract [15]**	0.251	0.374	0.266	0.404	0.209
**Diffusion Model [31]**	0.380	0.476	0.899	0.456	0.598

**Table 4:** Segmentation and ternary scores for RA on the single-annotation validation set of 30 FAF images. Best scores are drawn bold and underlined, second-best scores are drawn bold, ″ indicates scores are the same as for mean U-Net.

	Segmentation Task		Ternary Task		
	DcS	${\mathrm{mDcS}}_{\mathrm{AE}}$	${\mathrm{precision}}_{C}$	${\mathrm{recall}}_{\mathrm{CP}\to C}$	${\mathrm{DcS}}_{\mathrm{CP}}$
**Ensemble**	0.497	0.677	**1**	0.754	**0.679**
**Sub-Ens_10_**	**0.630**	**0.779**	0.940	**0.880**	**0.672**
**Sub-Ens_5_**	**0.628**	**0.787**	0.797	**0.791**	0.657
**Sub-Ens_3_**	0.559	0.717	0.679	0.662	0.620
**mean U-Net**	0.187	0.240	**1**	0.403	0.387
**mean + var U-Net**	″	″	0.917	0.370	0.394
**one U-Net**	0.501	0.622	0.832	0.512	0.634

**Table 5:** Segmentation and ternary scores for HF on the single-annotation test set of 60 FAF images. Best scores are drawn bold and underlined, second best scores are drawn bold, ″ indicates scores are the same as for mean U-Net.

	Segmentation Task		Ternary Task		
	DcS	${\mathrm{mDcS}}_{\mathrm{AE}}$	${\mathrm{precision}}_{C}$	${\mathrm{recall}}_{\mathrm{CP}\to C}$	${\mathrm{DcS}}_{\mathrm{CP}}$
**Ensemble**	0.466	0.587	**0.902**	**0.606**	**0.538**
**Sub-Ens_10_**	**0.494**	**0.631**	0.740	**0.631**	0.485
**Sub-Ens_5_**	0.482	0.616	0.778	0.590	0.526
**Sub-Ens_3_**	**0.485**	**0.645**	0.704	0.590	0.517
**mean U-Net**	0.409	0.521	**0.976**	0.573	**0.529**
**mean + var U-Net**	″	″	0.774	0.452	0.419
**one U-Net**	0.438	0.556	0.711	0.470	0.451
**H-Extract [15]**	0.203	0.320	0.240	0.353	0.168
**Diffusion Model [31]**	0.163	0.194	0.875	0.191	0.532

**Table 6:** Segmentation and ternary scores for RA on the single-annotation test set of 55 FAF images. Best scores are drawn bold and underlined, second best scores are drawn bold, ″ indicates scores are the same as for mean U-Net.

	Segmentation Task		Ternary Task		
	DcS	${\mathrm{mDcS}}_{\mathrm{AE}}$	${\mathrm{precision}}_{C}$	${\mathrm{recall}}_{\mathrm{CP}\to C}$	${\mathrm{DcS}}_{\mathrm{CP}}$
**Ensemble**	0.171	0.240	**1**	0.300	**0.272**
**Sub-Ens_10_**	**0.203**	0.290	**0.836**	**0.403**	0.267
**Sub-Ens_5_**	0.202	**0.298**	0.666	**0.333**	0.255
**Sub-Ens_3_**	**0.221**	**0.320**	0.569	0.280	0.241
**mean U-Net**	0.088	0.109	1	0.202	0.159
**mean + var U-Net**	″	″	433	203	157
**one U-Net**	0.141	0.202	0.439	0.163	**0.274**

**Table 7:** Pair-wise agreement on HF between the medical experts, the proposed network ensemble, light-weight sub-ensembles and single U-Nets. Agreement is measured in $mDcS_{AE}$ (black) and DcS (gray) scores averaged over nine FAF images. Expert 1* denotes segmentations of Expert 1 several months after the original annotations. For “one U-Net” the network with the highest DcS on the validation dataset was chosen out of the whole ensemble. The according results for RA are depicted in Table 8.

	Expert 1		Expert 2		Expert 3		Expert 4		Expert 1*		Ensemble	
**Expert 1**	1	1										
**Expert 2**	0.82	0.69	1	1								
**Expert 3**	0.76	0.65	0.78	0.67	1	1						
**Expert 4**	0.95	0.75	0.79	0.64	0.76	0.63	1	1				
Expert 1*	0.85	0.72	0.94	0.80	0.76	0.67	0.83	0.66	1	1		
**Ensemble**	0.86	0.73	0.91	0.76	0.76	0.64	0.86	0.68	0.96	0.81	1	1
**Sub-Ens_10_**	0.86	0.73	0.89	0.75	0.76	0.64	0.85	0.68	0.94	0.79	0.98	0.94
**Sub-Ens_5_**	0.85	0.70	0.86	0.71	0.75	0.62	0.84	0.66	0.91	0.76	0.96	0.90
**Sub-Ens_3_**	0.83	0.68	0.86	0.71	0.74	0.60	0.83	0.64	0.92	0.76	0.96	0.88
**mean U-Net**	0.85	0.71	0.92	0.76	0.75	0.62	0.86	0.67	0.93	0.81	0.97	0.93
**one U-Net**	0.83	0.71	0.89	0.75	0.74	0.62	0.83	0.68	0.93	0.78	0.98	0.90

**Table 8:** Agreement on RA measured in mean Dice score over nine FAF images. For details, see Table 7.

	Expert 1		Expert 2		Expert 3		Expert 4		Expert 1*		Ensemble	
**Expert 1**	1	1										
**Expert 2**	0.50	0.39	1	1								
**Expert 3**	0.44	0.30	0.55	0.37	1	1						
**Expert 4**	0.49	0.45	0.31	0.27	0.18	0.14	1	1				
Expert 1*	0.58	0.44	0.73	0.52	0.66	0.47	0.33	0.28	1	1		
**Ensemble**	0.77	0.68	0.42	0.26	0.32	0.22	0.45	0.41	0.37	0.27	1	1
**Sub-Ens_10_**	0.75	0.69	0.44	0.28	0.34	0.24	0.43	0.39	0.38	0.29	0.96	0.83
**Sub-Ens_5_**	0.73	0.65	0.42	0.28	0.27	0.18	0.45	0.40	0.35	0.26	0.93	0.80
**Sub-Ens_3_**	0.61	0.50	0.47	0.30	0.33	0.17	0.36	0.28	0.39	0.27	0.76	0.59
**mean U-Net**	0.70	0.60	0.31	0.21	0.23	0.15	0.69	0.61	0.33	0.24	0.75	0.67
**one U-Net**	0.65	0.59	0.32	0.23	0.25	0.16	0.36	0.33	0.31	0.23	0.84	0.73

**Table 9:** Ternary task performance for HF as means over nine FAF images. The ground truth was created from all five available expert annotations (Expert 1* denoting annotations of Expert 1 several months after the original annotations) via the method explained in Figure 5. Hence, the expert scores are only given as a reference and cannot necessarily be directly compared to the scores of the proposed methods (see $recall_{CP\to C}$ ), as they are calculated for ground truth they influenced. “Ensemble all *C*” denotes scores when mapping the ensembles *C* and *P* predictions all to *C*. The “all *P*” denotes the theoretical scores for predicting every pixel as *P*. Best scores for the proposed methods are drawn bold and underlined, second-best scores are drawn bold.

	${\mathrm{precision}}_{C}$	${\mathrm{precision}}_{C\to \mathrm{CP}}$	${\mathrm{recall}}_{\mathrm{CP}}$	${\mathrm{recall}}_{\mathrm{CP}\to C}$	${\mathrm{DcS}}_{\mathrm{CP}}$
**Expert 1**	0.57	0.93	0.72	1	0.85
**Expert 2**	0.56	0.91	0.85	1	0.88
**Expert 3**	0.58	0.77	0.74	1	0.71
**Expert 4**	0.69	0.96	0.62	1	0.79
Expert 1*	0.55	0.93	0.86	1	0.89
**Ensemble**	**0.71**	**0.97**	**0.88**	**0.99**	**0.81**
**Sub-Ens_10_**	**0.68**	**0.96**	**0.88**	**0.99**	0.74
**Sub-Ens_5_**	**0.68**	**0.96**	**0.88**	**0.99**	0.72
**Sub-Ens_3_**	0.63	0.93	0.86	0.97	0.77
**mean U-Net**	0.59	0.92	**0.90**	**1**	**0.80**
**mean + var U-Net**	0.57	0.82	0.87	0.98	**0.81**
**one U-Net**	0.57	0.88	0.79	0.96	0.79
**Ensemble all *C***	0.45	0.78	0.88	0.99	0.81
**all *P***	-	-	1	1	0.17

**Table 10:** Ternary task performance for RA as means over nine FAF images. For details, see Table 9. Missing precision scores are due to no area being predicted as *C*.

	${\mathrm{precision}}_{C}$	${\mathrm{precision}}_{C\to \mathrm{CP}}$	${\mathrm{recall}}_{\mathrm{CP}}$	${\mathrm{recall}}_{\mathrm{CP}\to C}$	${\mathrm{DcS}}_{\mathrm{CP}}$
**Expert 1**	0.51	0.88	0.52	1	0.78
**Expert 2**	0.21	0.56	0.85	1	0.59
**Expert 3**	0.37	0.88	0.58	1	0.72
**Expert 4**	0.28	0.84	0.26	1	0.66
Expert 1*	0.22	0.79	0.74	1	0.76
**Ensemble**	-	-	**0.59**	**1**	**0.55**
**Sub-Ens_10_**	1	1	**0.68**	**1**	**0.45**
**Sub-Ens_5_**	1	0.72	0.57	**1**	0.43
**Sub-Ens_3_**	0.99	0.71	0.58	**1**	0.44
**mean U-Net**	0.85	0.94	0.31	0.99	0.29
**mean + var U-Net**	0.12	0.60	0.30	0.76	0.34
**one U-Net**	0.13	0.66	0.27	0.50	0.39
**Ensemble all *C***	0.22	0.56	0.59	1	0.55
**all *P***	-	-	1	1	0.09

**Table 11:** Runtimes in seconds for the different ensembles measured on an NVIDIA Titan Xp GPU with an Intel Core i7-4790K CPU with 4.00 GHz. The times shown here are the means over three runs with ± showing the standard deviation while the time/model ratio is given in gray and in brackets. For the diffusion model, we present the runtime of a single forward pass.

Method	Models	Loading Weights	First Image	Each Additional Image
**Ensemble**	100	340.33 ± 15.71 (3.40)	48.73 ± 1.61 (0.49)	3.29 ± 0.33 (0.033)
**Sub-Ens_10_**	10	24.71 ± 1.94 (2.47)	5.73 ± 0.08 (0.57)	0.30 ± 0.04 (0.030)
**Sub-Ens_5_**	5	9.23 ± 0.16 (1.85)	3.28 ± 0.07 (0.66)	0.18 ± 0.04 (0.035)
**Sub-Ens_3_**	3	5.65 ± 0.15 (1.88)	2.40 ± 0.03 (0.80)	0.12 ± 0.02 (0.039)
**one U-Net**	1	2.63 ± 0.27 (2.63)	1.41 ± 0.03 (1.41)	0.07 ± 0.02 (0.066)
**Diffusion Model [31]**	-	2.73 ± 0.03	-	54.36 ± 0.94

## Data Availability

The data are currently not publicly available.

## Supplementary Materials

The following supporting information can be downloaded at: https://www.mdpi.com/article/10.3390/jimaging10050116/s1, The accompanying supplementary contains in Section S1 additional evaluation regarding the correlation [63,64] of training set and prediction similarity. Included in this section is Figure S1 visualizing this correlation. Section S2 of the supplementary analyzes and depicts in Figure S2 the validation performance for different annotation strategies and training set size. Section S3 of the supplementary analyzes and depicts in Figures S3 and S4, and Table S1 the robustness of our segmentation models against image noise [65].

## Abbreviations

The following abbreviations are used in this manuscript:

HF	(diffuse) hyperfluorescence
RA	reduced autofluorescence (also hypofluorescence)
FAF	fundus autofluorescence
CSCR	central serous chorioretinopathy
GA	geographic atrophy
AMD	age-related macular degeneration
RPE	retinal pigment epithelium
qAF	quantitative autofluorescence
AI	artificial intelligence
CNN	convolutional neural network
DcS	Dice score
IoU	intersection over union
TP	true positive
TN	true negative
FP	false positive
FN	false negative
EE	edge error
AE	area error
MDP	Maximum Diversity Problem
MMDP	Max–Min Diversity Problem
MSE	mean squared error
HE	HyperExtract algorithm [15]
GPU	graphics processing unit
CPU	central processing unit

## Appendix A

This appendix shows in Figure A1, Figure A2, Figure A3 performance differences of the network ensemble between validation and test data i.r.t. HF annotation size and according comparisons between the test and validation dataset. The appendix further shows in Figure A4, Figure A5, Figure A6, Figure A7 several points of analysis regarding the HyperExtract baseline [15] for HF segmentation. Figure A8 depicts qualitative results for the diffusion model by [31].
