# Supplementary material for: When Two Eyes Don’t Suffice—Learning Difficult Hyperfluorescence Segmentations in Retinal Fundus Autofluorescence Images via Ensemble Learning
Source: J Imaging. 2024 May 9;10(5):116. doi: 10.3390/jimaging10050116 (PMC11122615; doi:10.3390/jimaging10050116)
Supplement: Supplementary file 1 [file jimaging-10-00116-s001.zip › jimaging-2991531-supplementary.pdf]

Supplementary Materials: When Two Eyes Don't Suffice—Learning Difficult Hyperfluorescence Segmentations in Retinal Fundus Autofluorescence Images via Ensemble Learning

Monty Santarossa, Tebbo Tassilo Beyer, Amelie Bernadette Antonia Scharf, Ayse Tatli, Claus von der Burchard, Jakob Nazarenus, Johann Roider and Reinhard Koch

1. Training—Correlation between Training Sets and Prediction Similarity

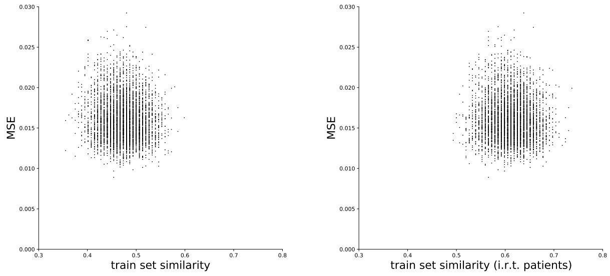

(a) over unique images **(b)** over unique patients

Figure S1. The pair-wise correlation between the similarity of HF predictions on the validation data (measured in MSE) and the similarity of their training data (ratio of shared images (a) or patients (b) compared to the training dataset size of 152 samples) for the individual segmentation networks of our ensemble. The Pearson correlation coefficients [1] for (a) and (b) are -.063 and -.041, the Spearman rank correlation coefficients [2] are -.044 and -.037, respectively, indicating no significant correlation.

Since for our sub-ensemble we sample models based on (dis-)similarity, we analyze in Figure S1 whether we can predict prediction similarity between two models based on the similarity of their training subsets. This does not seem to be the case, as low Pearson [1] and Spearman [2] correlation coefficients indicate.

2. Annotation Costs

In this work, we analyze the size of HF annotations as an indicator for the model's performance. While in reality, the selection of data would have to happen from raw FAF images, we simulate the effect of different selection strategies by considering four different sampling strategies from our pool of annotated data: 1) *max*, i.e. choosing the b images with the largest HF area. 2) *min*, i.e. choosing the b images with the smallest HF area (empty images are omitted). 3) *med*, i.e. choosing the sample with a median HF area and selecting the next $\lfloor \frac{b}{2} \rfloor$ samples with a smaller and the next $\lceil \frac{b}{2} \rceil$ samples with a larger HF area. 4) *mix*, i.e. selecting from the whole range of HF areas with equal step size between samples. Note that *mix* is the only sampling strategy of the four, where sets for smaller b do not have to be subsets of sets for larger b. Finally, for comparison, we also use 5) *all* of our available data.

Next, we examine how to best spend the budget (e.g. time/cost) available for annotation. Given a set of suitable FAF images and a budget b denoting the number of images we can annotate, it is our goal to select samples such as to optimize the expected segmentation score of the model trained on this data.

In order to widen our pool of available data for this analysis, we combined our training and test set into one larger training set and performed evaluation solely on our validation data of 212 images. We analyzed budgets of 64, 96, 128, 160, and 192, training five models for each sampling strategy and budget.

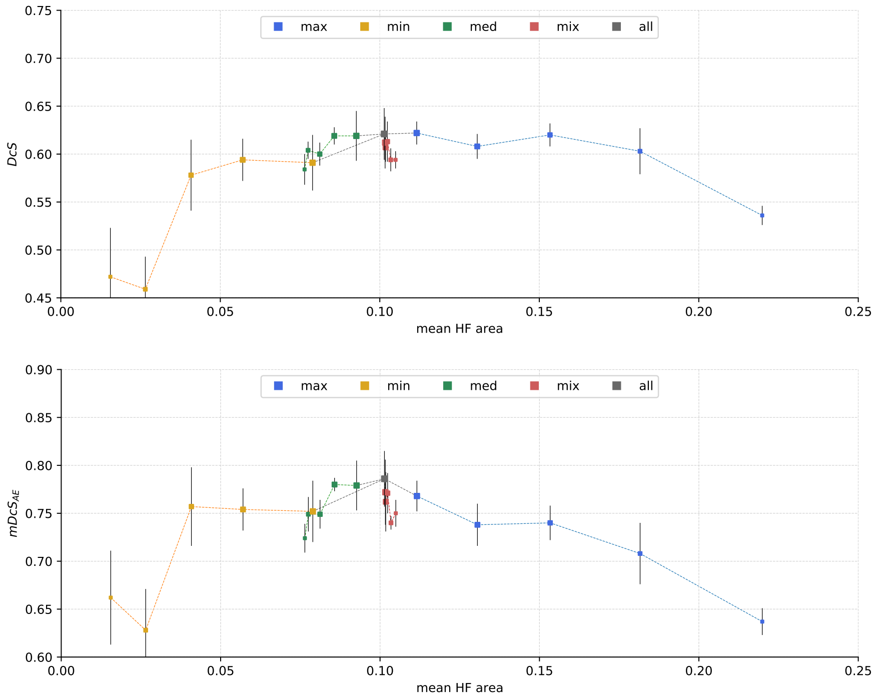

Figure S2. Single model segmentation performance measured in Dcs (top) and $mDcS$ (bottom) on the single annotation validation dataset depending on the training set size and the mean annotated HF area per image. Training set sizes are 64, 96, 128, 160, 192, and *all* (=212, only gray), and are visualized by the size of the square markers (bigger marker indicates larger training set). In addition, results for the training sets of neighboring size are connected by dashed lines (i.e., 96 to 64 and 128). The colors indicate training set sampling approaches. Depicted are the mean scores and standard deviation error bars for five models.

The corresponding segmentation results are depicted in Figure S2. Notably, *min* is not a good sampling strategy, performing worst for each budget. *max* seems to be a good sampling strategy, considering DcS, reaching the highest score for the maximum budget of $b = 192$. Looking at $mDcS_{AE}$, however, reveals that false segmentations for *max* largely consist of area errors as might be expected from this approach being trained on large annotations, leading to comparatively low $mDcS_{AE}$ scores. *mix* might be a good sampling strategy for lower budgets $b \leq 128$, but does not yield much improvement for the larger budgets. Finally, *med* yields good results for smaller budgets and the best results for larger budgets. For all sampling strategies, larger budgets, in most cases, lead to better segmentation performance.

3. Robustness against Noise

We analyze the relationship between the segmentation performance and image noise in regard to two aspects: 1) The correlation between DcS_{AE} and image noise on our data as is. 2) The influence of a lower peak signal-to-noise ratio (PSNR) when we manually degrade image quality by adding Gaussian noise.

3.1. Correlation between Segmentation Score and Image Noise

We estimate image noise by the approach of J. Immerkær [3]. Figure S3 plots for several methods HF DcS_{AE} scores over image noise in the validation and test dataset. The corresponding correlations scores are given in Table S1.

From the results, we see that correlation coefficients are low (maximum absolute of 0.251 for Pearson and 0.237 for Spearman) and that generally positive coefficients for validation data would indicate that segmentations improve with a higher noise. Taking

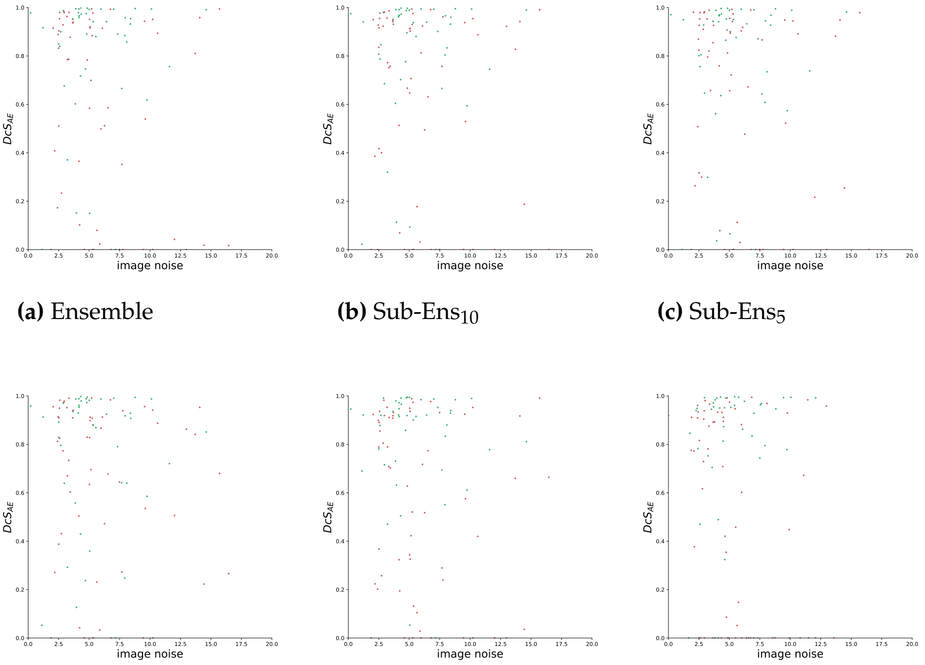

(a) Ensemble **(b)** Sub-Ens$_{10}$ **(c)** Sub-Ens$_5$

(d) Sub-Ens$_3$ **(e)** one U-Net **(f)** mean U-Net

Figure S3. DcS_{AE} scores over image noise [3] for each image in the validation (green) and test (red) dataset for our proposed ensembles (a to d) and U-net baselines (e and f). The corresponding correlation scores are given in Table S1. Please note that the graph for "mean + variance U-Net" would be identical to the depicted graph for mean U-Net (f).

Table S1. The Pearson [1] and Spearman [2] correlations coefficients for DcS_{AE} and image noise [3], as depicted in Figure S3.

	Pearson correlation			Spearman correlation		
method	validation	test	both	validation	test	both
**Ensemble**	0.135	−0.251	−0.163	0.122	−0.228	−0.093
**Sub−Ens$_{10}$**	0.133	−0.143	−0.082	0.129	−0.131	−0.043
**Sub−Ens$_5$**	0.123	−0.218	−0.134	0.150	−0.218	−0.081
**Sub−Ens$_3$**	0.150	−0.134	−0.091	0.042	−0.180	−0.118
**mean U−Net**	0.116	−0.250	−0.160	0.192	−0.237	−0.064
**one U−Net**	−0.060	−0.155	−0.152	0.006	−0.166	0.110

into account the generally low noise values in our data as well (see Figure S3), we conclude that on our given data noise does not significantly affect segmentation quality.

3.2. Influence of Peak-Signal-to-Noise-Ratio on Segmentation

Since true signal-to-noise calculations requires signals, i.e., images, with no noise, which we do not possess, we do estimations by starting from images with very little noise and successively adding stronger noise for comparison.

From data shown in Figure S3a, we select 10 images with high DcS_{AE} and little noise. For this, we sort each image I by the score $sc(I) = DcS_{AE}(I) \cdot (1 - \frac{noise(I)}{noise_{max}})$, where $DcS_{AE}(I)$ is the ensemble's DcS_{AE} score for I, $noise_I$ is the noise for I and $noise_{max}$ is the maximum noise over all images. The 10 images with the highest score get chosen for further analysis.

By considering these images as pseudo-perfect images (i.e. images with no noise), we can calculate a peak signal-to-noise ratio $PSNR = 20 \log_{10} \frac{255}{\sqrt{MSE}}$, where 255 is the

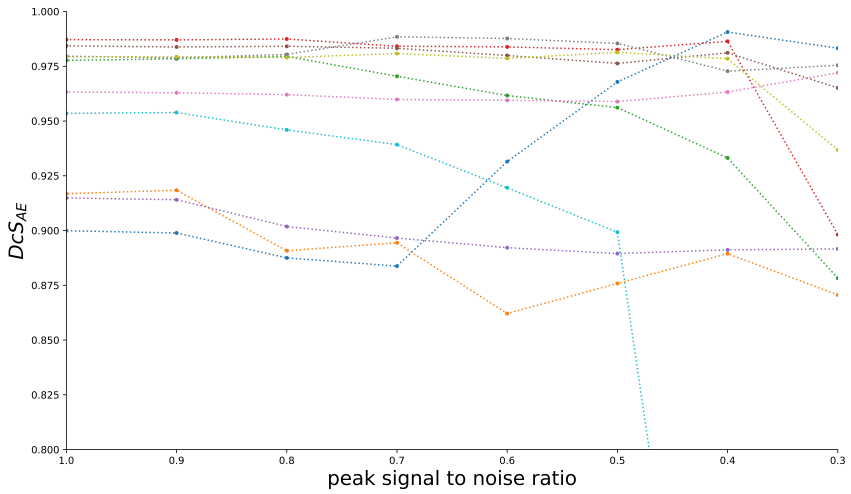

Figure S4. HF segmentation scores of the full ensemble, depending on peak signal-to-noise ratio for 10 images to which Gaussian noise was successively applied. Different colors indicate different images.

maximum possible pixel intensity in the image and MSE is the mean squared error between the pseudo-perfect image and the noisy image. We add to the 10 selected pseudo-perfect

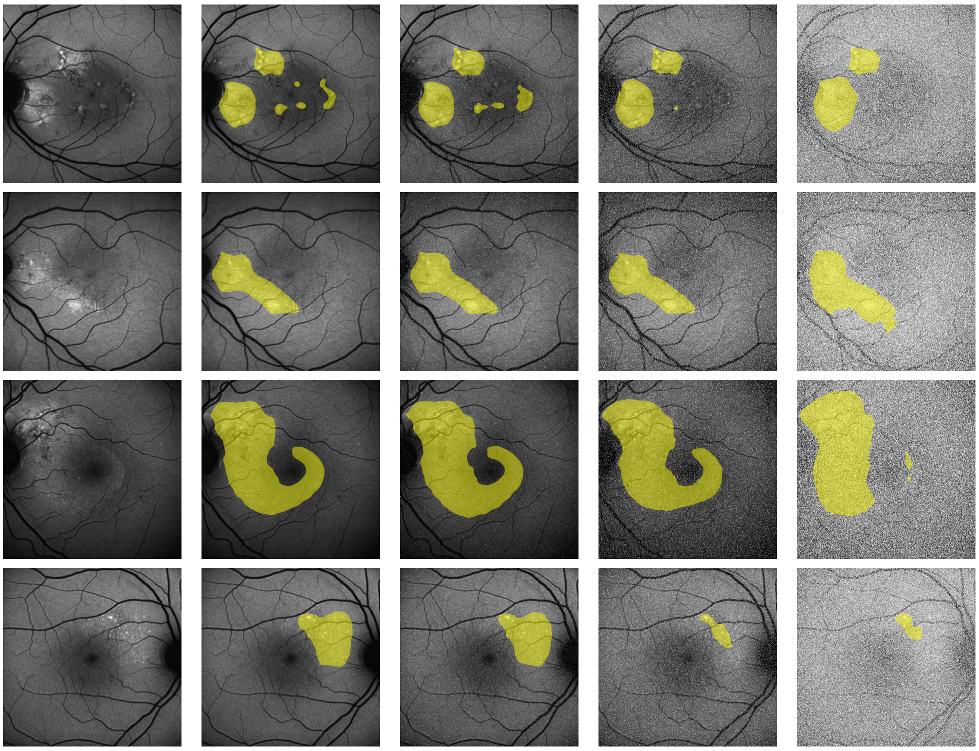

(a) Input **(b)** PSNR = 1.0 **(c)** PSNR = 0.7 **(d)** PSNR = 0.4 **(e)** PSNR = 0.3

Figure S5. (Images are best viewed zoomed in). HF segmentation performance of the full ensemble depending on images (a), to which Gaussian noise was applied PSNR (c-e). For comparison, original segmentations are given in (b), where no additional noise was applied.

images Gaussian noise such that we achieve PSNRs of [1.0, 0.9, . . . , 0.4, 0.3] and let our full ensemble predict HF on these noisy images.

Resulting DcS_{AE} are plotted in Figure S4. Examples for noisy images and resulting segmentation are given in Figure S5. From the data shown, we see that our ensemble is highly robust against noise for $PSNR \geq 0.4$ and generally robust for $PSNR \geq 0.3$. Significant drops in DcS_{AE} can only be observed on three images and very low $PNSR$ (light blue line in Figure S4 at $PNSR = 0.4$, red and green line for $PNSR = 0.3$).

References

1. Kirch, W., Ed., Pearson's Correlation Coefficient. In *Encyclopedia of Public Health*; Kirch, W., Ed.; Springer Netherlands: Dordrecht, 2008; pp. 1090–1091. https://doi.org/10.1007/978-1-4020-5614-7_2569.
2. Dodge, Y., Spearman Rank Correlation Coefficient. In *The Concise Encyclopedia of Statistics*; Springer New York: New York, NY, 2008; pp. 502–505. https://doi.org/10.1007/978-0-387-32833-1_379.
3. Immerkaer, J. Fast noise variance estimation. *Computer vision and image understanding* **1996**, *64*, 300–302.

Disclaimer/Publisher's Note: The statements, opinions and data contained in all publications are solely those of the individual author(s) and contributor(s) and not of MDPI and/or the editor(s). MDPI and/or the editor(s) disclaim responsibility for any injury to people or property resulting from any ideas, methods, instructions or products referred to in the content.
